# Phenolic Transfer and Dissolved-Oxygen Dynamics in Different White Wines Treated with Two *Quercus alba* Wood Samples with Contrasting Grain Widths

**DOI:** 10.3390/foods15183176

**Published:** 2026-09-08

**Authors:** Ana María Martínez-Gil, Maria del Alamo-Sanza, Clara Campo Hernández-Sampelayo, Ignacio Nevares

**Affiliations:** 1 Department of Analytical Chemistry, UVaMOX Group, University of Valladolid, 34001 Palencia, Spain; anamaria.martinez.gil@uva.es (A.M.M.-G.); clara.campo@estudiantes.uva.es (C.C.H.-S.); 2 Department of Agricultural and Forestry Engineering, UVaMOX Group, University of Valladolid, 34001 Palencia, Spain; ignacio.nevares@uva.es

**Keywords:** *Quercus alba*, Verdejo, Albillo, Rufete, Puesta en Cruz, white wine, wood–wine interaction, phenolic transfer

## Abstract

American oak is widely used in enology, but its interactions with white wine and the role of wood properties remain poorly understood. In this study, oxygen dynamics and changes in the phenolic composition of wines made from the Verdejo, Albillo, Rufete, and Puesta en Cruz varieties were evaluated over 35 days of contact with toasted pieces from two *Quercus alba* wood samples with contrasting grain widths. Untreated control wines and a model wine treated separately with each wood were also included. Wood density and impregnation were determined, dissolved-oxygen partial pressure was monitored noninvasively, and routine enological parameters, spectrophotometric indices, and low-molecular-weight phenolic compounds were analyzed in the final wines. Contact with wood modified the net dissolved-oxygen profiles, although the relative effects of the two contrasting wood samples depended on the wine matrix. The fine-grain wood tested showed greater impregnation and generally produced higher phenolic indices, larger changes in color, and higher concentrations of several wood-derived compounds. Overall, the results show that the properties of the two wood samples and wine composition jointly shape the response of white wines to oak contact.

## 1. Introduction

During fermentation or aging, contact between wine and wood triggers physical and chemical interactions that shape wine evolution. These include the extraction of wood-derived compounds, mainly ellagitannins, low-molecular-weight phenolic compounds, and volatile compounds, as well as processes related to oxygen release and consumption [1]. The wine’s response to these processes depends on both the characteristics of the wood and the initial composition of the wine, resulting in distinct chemical and sensory profiles.

Among the species used in enology, oak is the predominant wood used in winemaking and aging. The International Organisation of Vine and Wine (OIV) [2] authorizes the use of oak (*Quercus*) and chestnut for cooperage and restricts enological wood pieces to species of the genus *Quercus* [3]. Traditionally, the most widely used species have been the European oaks *Quercus petraea* and *Quercus robur*, as well as American oak *Quercus alba*, whose anatomical and compositional differences shape their behavior during contact with wine.

*Quercus alba* is one of the most widely used species in enology because of its availability, high processing yield, and the compounds it contributes to wine. The abundance of tyloses allows the wood to be sawn without compromising its watertightness, resulting in a higher material yield than that of European oaks, which generally require splitting. American oak is also characterized by its density, mechanical strength, porosity, and permeability [4,5]. In addition, *Q. alba* is characterized by a distinct compositional profile, with higher concentrations of lactones and lower ellagitannin contents than European oaks, as well as a higher proportion of gallotannins [4].

Aging in wood has traditionally been associated with red wines, whereas white wines have generally been marketed as young, fresh styles intended for early consumption. However, changing consumer preferences toward more complex and distinctive wines have driven interest in fermentation and aging in contact with wood [1,6,7]. Consequently, both the use of wood in wineries and research into its effects on the chemical and sensory composition of white wines have increased [1,6,7,8,9,10,11,12,13]. Oxygen management is particularly relevant because the oxidative stability of white wines depends on their phenolic composition, SO_2_ content, the presence of metals, and other matrix components that participate in oxidation reactions.

Since the use of wood pieces in enology was authorized in 2006 (Commission Regulation (EC) No. 1507/2006) [14], they have become established as an alternative to barrel aging because of their lower cost, ease of use, and shorter aging time [6]. In white wines, this alternative is particularly relevant because it allows more flexible control of the wine–wood interaction and oxygen supply through micro-oxygenation (MOX), a critical factor given the greater susceptibility of these wines to oxidation [15]. Nevertheless, most studies have focused on red wines, and knowledge of how alternative wood products behave in different white wines remains limited, especially regarding conditions of use (toasting, dose, contact time) and oxygen management during aging [1]. In this context, several studies have addressed the use of wood pieces in white winemaking, primarily in Chardonnay [8,13,15,16,17]. Other white varieties have also been studied, such as Verdejo, Sauvignon Blanc, Malvazija Istarska, Encruzado, and Listán, with different wood species [1,7,9,12,13,18,19,20]. Overall, these studies indicate that the initial composition of the wine can modify the extraction, transformation, and stability of wood-derived compounds. However, most studies evaluate a single variety, leaving little information on the comparative response of different wine matrices under identical wood-contact conditions.

At the same time, the wine sector is showing growing interest in the revival and valorization of minority white grape varieties, driven by demand for more diverse and distinctive wines and by the potential of these varieties to adapt to climate change; some ripen later and show greater disease resilience [21,22]. Within this framework, wine-growing regions such as Castilla y León represent a particularly interesting model, as widely established varieties such as Verdejo coexist with a significant group of native minority varieties now receiving renewed attention [23,24]. This creates an opportunity to examine the interaction between varietal matrix and aging in wood within a single experimental framework.

Although the effects of factors such as wood species, toasting level, dose, and piece size have been widely studied, little information is available on how *Quercus alba* wood samples with contrasting growth-ring widths perform when used as alternative oak products. It also remains unclear whether differences between these two wood samples consistently translate into differences in initial oxygen release, wood impregnation, and the transfer of phenolic compounds, or to what extent the initial composition of the wine modulates these responses.

Therefore, in the present study, two toasted *Q. alba* wood samples with contrasting ring widths, used at the same dose and contact time, were compared across four monovarietal white wines, alongside the corresponding controls and a model wine treated with each wood. This design allowed the joint effects of wood properties and wine matrix on dissolved-oxygen dynamics and phenolic composition during wine–wood contact to be evaluated.

## 2. Materials and Methods

### 2.1. Woods

The *Quercus alba* wood was supplied by the INTONA cooperage (Navarra, Spain). The two staves used in this study were selected from a larger set of 214 Q. alba staves previously characterized for their macrostructural and anatomical features using image analysis [5]. Based on these data, two staves with markedly contrasting anatomical features were selected for the present study. One stave had wide growth rings (5.1 mm) whereas the other had narrow growth rings (1.1 mm); they were designated as wide-grain (WG) and fine-grain (FG), respectively. Thus, WG and FG refer here to the two specific staves selected for this study. The selected staves were then cut into pieces approximately 12.5 cm in length × 2.7 cm in width × 1 cm in thickness, with the length adjusted to the original width of the stave to obtain wood fragments suitable for addition to the wine while maintaining the target wood-to-wine ratio throughout the contact trial. The pieces were then toasted by gradually raising the temperature to 210 °C and holding it for 35 min in an industrial-scale convection oven, using supports specially adapted to the oven trays for alternative wood products (model 9110-0212; BINDER APT-COM., New York, NY, USA).

### 2.2. Wines

The monovarietal wines were produced during the 2025 harvest at the Rueda Oenological Station (Valladolid, Spain) using four *Vitis vinifera* L. varieties: Verdejo, Albillo, Rufete, and Puesta en Cruz. The general chemical characteristics of the four white wine matrices are shown in Table 1.

### 2.3. Experimental Design

The wood-to-wine volume ratio was kept constant in all experimental units and set to match that of a 225 L barrel (45 cm^3^/L). Each 750 mL bottle received a wood piece with a volume of 33.5 cm^3^.

The experimental design included two wood samples (WG and FG) and four white wine matrices (varieties Verdejo, Albillo, Rufete, and Puesta en Cruz) (Table 2). Each wine–wood combination was prepared in triplicate, resulting in three replicates per wine matrix and wood treatment and a total of 24 experimental units (2 wood samples × 4 varieties × 3 replicates). In addition, control wines without wood were included for each matrix, with two replicates per variety, resulting in 8 experimental units (4 varieties × 2 replicates). Finally, a model wine consisting of an ethanol:water solution (15:85) at pH 3.5 was tested separately with each wood sample, with three replicates per wood treatment, resulting in 6 additional experimental units (2 wood samples × 3 replicates). Thus, the study comprised 38 experimental units in total. All treatments were carried out in 750 mL transparent glass bottles with oxygen-tight closures.

The bottles were filled with wine, leaving no headspace, and were kept sealed for 35 days in a temperature-controlled room at 15.5 ± 0.5 °C. During this period, they were shaken once daily to promote the release of oxygen and compounds from the wood to the wine. At the end of the contact period, the wood was removed and weighed to quantify the impregnation of each piece in absolute terms (g) and as a percentage of its initial dry weight (an indicator of the degree of impregnation), after which the corresponding analyses were performed.

Dissolved-oxygen monitoring. All bottles were equipped with a PSt6 luminescent optical sensor (PreSens GmbH, Regensburg, Germany), attached beforehand to the inner wall of the bottles and calibrated according to the manufacturer’s protocol. The sensors enabled hourly, noninvasive oxygen measurements with temperature compensation. Signals were recorded using OXY-4 SMA and Fibox 4 Trace instruments (PreSens GmbH, Regensburg, Germany).

### 2.4. Analysis of Basic Chemical Parameters

The wines were analyzed initially (Table 1) and after 35 days of contact with each wood. The routine physicochemical parameters—pH, total acidity (g/L of tartaric acid), volatile acidity (g/L of acetic acid), free sulfur dioxide (F-SO_2_, mg/L), and total sulfur dioxide (T-SO_2_, mg/L)—were measured following the methods of the International Organisation of Vine and Wine [25].

All experimental units were analyzed in duplicate.

### 2.5. UV–Vis Spectrophotometric Analysis

The absorbance spectra of the wines were recorded over the range 350–780 nm at 5 nm intervals. Color intensity (CI) was expressed as the absorbance at 420 nm (A420) [26]. The total polyphenol index (TPI) was determined according to the International Organisation of Vine and Wine [25]. Tartaric esters and flavonols were determined according to Mazza et al. [27]. Finally, total tannins (TAN, expressed as g/L of cyanidin chloride) were analyzed using the method of Riberéau-Gayon and Stonestreet [28]. All measurements were performed using quartz cuvettes with a 1 cm path length and a PerkinElmer Lambda 25 UV–visible spectrophotometer (Waltham, MA, USA). All experimental units were analyzed in duplicate.

### 2.6. Analysis of Phenolic Compounds in Wood and Wine

#### 2.6.1. Wood Extract Preparation

The toasted wood was ground, and a sufficiently large batch was homogenized to ensure representative sampling. A 2 g portion was then taken for each replicate. Extraction was carried out using 20 mL of a hydroalcoholic solution (water:methanol, 85:15, *v*/*v*). The mixture was shaken by hand for 1 min, vortexed for 1 min, left to macerate for 24 h at room temperature, and finally filtered. The filtrate was collected in a 20 mL volumetric flask using an in-line double-filtration system consisting of a fast filter placed above a slow filter. The solution was then made up to volume with the hydroalcoholic solution. Each wood sample was extracted in triplicate, yielding a total of six extracts. The concentrations determined in the wood extracts represent the fraction recovered under the extraction conditions described above.

#### 2.6.2. Analysis of Individual Phenolic Compounds

Phenolic compounds were analyzed using a high-performance liquid chromatography system with diode array detection (HPLC–DAD; Agilent 1100, Palo Alto, CA, USA), as described by Del Barrio-Galán et al. [29]. Chromatographic separation was performed on a reversed-phase Zorbax SB-C18 column (250 × 4.6 mm i.d., 3.5 µm particle size) (Agilent, Barcelona, Spain).

Wood extracts were filtered through 0.45 µm nylon membrane filters, whereas wine samples were centrifuged at 4000 rpm for 10 min and subsequently filtered through nylon membranes. Elution was carried out at a flow rate of 0.6 mL/min and a column temperature of 25 °C. Solvent A consisted of acetic acid/water (2:98, *v*/*v*), and solvent B consisted of acetic acid/acetonitrile/water (2:20:78, *v*/*v*/*v*). The gradient program was as follows: 0–25 min, 100% solvent A; 25–60 min, 75% solvent A and 25% solvent B; 60–100 min, 30% solvent A and 70% solvent B; and 100–135 min, 100% solvent B. The injection volume was 30 µL.

Diode array detection was performed over the range 200–400 nm. Quantification was carried out at 254 nm for protocatechuic acid and vanillic acid; 280 nm for gallic acid, syringic acid, vanillin, furfural, 5-methylfurfural, 5-hydroxymethylfurfural, catechin, and epicatechin; 320 nm for sinapic acid, trans-p-coumaric acid, ferulic acid, and syringaldehyde; and 340 nm for trans-caffeic acid. Compounds were identified by comparing their retention times and UV–visible spectra with those of authentic standards. Quantification was performed using seven-point calibration curves prepared from the corresponding standards, with satisfactory linearity for all compounds (R^2^ > 0.9967). The calibration range, retention time, detection wavelength, regression equation, and R^2^ values for each compound are provided in Supplementary Table S1. Results were expressed as µg/g of dry wood for the wood extracts and as mg/L for the wines.

Phenolic compounds for which commercial standards were not available were tentatively identified from their retention times and UV–visible spectra, following the method described by Pérez-Magariño et al. [30], and were quantified using the calibration curve of the most structurally similar available standard. These compounds included coniferaldehyde and sinapaldehyde (quantified as syringaldehyde), cis-coumaric acid and tyrosol (quantified as trans-coumaric acid), cis-coutaric acid, and trans-fertaric acid (quantified as trans-caffeic acid). Results for compounds quantified using alternative standards are expressed as equivalents of the standard used for quantification and should therefore be interpreted as semiquantitative values.

#### 2.6.3. Relative Transfer Index of Phenolic Compounds from Wood to Wine

The relative transfer index (RTI, %) normalizes the extraction achieved in each real wine to that achieved in the model wine treated with the same wood for the same period, thereby helping to distinguish the effect of the wine matrix from that of the wood itself. Because the same fine-grain (FG) and wide-grain (WG) wood samples were used in all cases, differences in RTI among wines reflect the composition of each wine and the accessibility of the compounds in each wood, rather than differences in their initial concentrations in the wood.

The relative transfer index (RTI) for each compound was calculated as:

(1) $$\mathrm{RTI}\;\left(\%\right)\;=\;100\;\cdot \left[\frac{C_{\mathrm{wine}+\mathrm{wood}}-C_{\mathrm{control}}}{C_{\mathrm{synthetic}+\mathrm{wood}}}\right]$$

where

- C_wine+wood_ is the final concentration of the compound in the wine treated with wood.
- C_control_ is the final concentration in the control wine without wood.
- C_synthetic+wood_ is the final concentration in the model wine treated with the same wood.

### 2.7. Statistical Analysis

Statistical analyses were performed using Statgraphics Centurion software (version 19.4.02; StatPoint, Inc., The Plains, VA, USA). Analytical duplicates were averaged within each independent experimental unit before statistical analysis. One-way analysis of variance (ANOVA) followed by Tukey’s honestly significant difference (HSD) test (*p* < 0.05) was used to test (i) differences among treatments (Control, WG and FG) within each wine matrix (lowercase letters) and (ii) differences among wine matrices within each treatment (Control, WG or FG) (uppercase letters). For comparisons among wood treatments within each wine variety, the degrees of freedom were 2 and 5 for the treatment and residual terms, respectively (total df = 7). For comparisons among wine varieties within WG and FG treatments, the degrees of freedom were 3 and 8 for the variety and residual terms, respectively (total df = 11), whereas for control wines, they were 3 and 4, respectively (total df = 7). A two-way ANOVA was performed to evaluate the effects of wood treatment, wine matrix, and their interaction on the measured parameters, with degrees of freedom of 2, 3, 6, 20, and 31 for wood treatment, variety, wood × variety interaction, residual error, and total, respectively. Pearson’s correlation analysis was used to assess relationships among variables. Principal component analysis (PCA) was performed to examine the separation of wood samples and wines based on their phenolic composition. Three PCA models were constructed: (i) using the wood samples, (ii) using all wine samples (controls and wines in contact with wood), and (iii) using only wines in contact with wood to facilitate separation between the WG and FG treatments.

## 3. Results and Discussion

### 3.1. Physical Properties of the Wood

**Figure 1:**
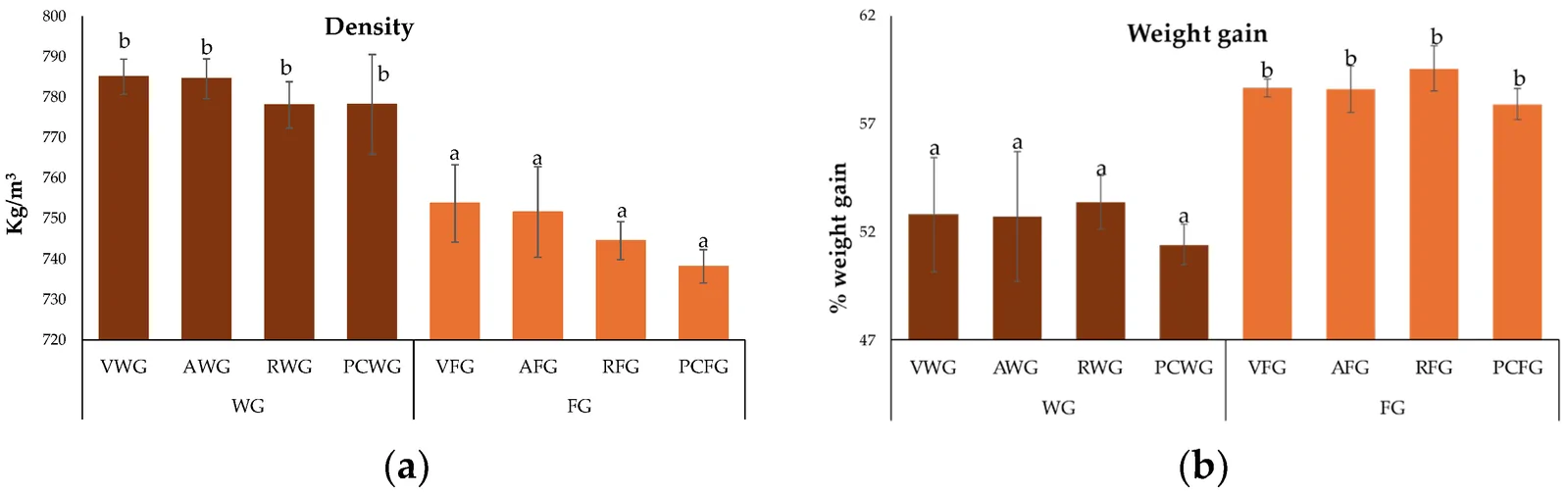
(**a**) Density (kg/m^3^) of wide-grain (WG) and fine-grain (FG) oak samples; (**b**) weight gain (%) after 35 days of contact with the wines (Verdejo, Albillo, Rufete, and Puesta en Cruz). Data are presented as mean ± standard deviation (SD), with *n* = 2 for control wines and *n* = 3 for wines treated with wood, where *n* represents independent experimental units. Different lowercase letters indicate significant differences between the WG and FG wood samples.

Figure 1b shows that the wide-grain (WG) pieces generally had a higher density than the fine-grain (FG) pieces, whereas the FG pieces showed greater weight gain after contact with the wine. This behavior indicated greater impregnation of the FG wood under the experimental conditions and may have favored the initial release of retained air and soluble compounds. Nevertheless, because the pieces were fully submerged—unlike wood in a barrel—their radial, tangential, and transverse faces were simultaneously exposed to wine; therefore, the effects of grain width could not be separated from those of density, porosity, piece size, and contact surface area.

### 3.2. Net Dissolved-Oxygen Dynamics

**Figure 2:**
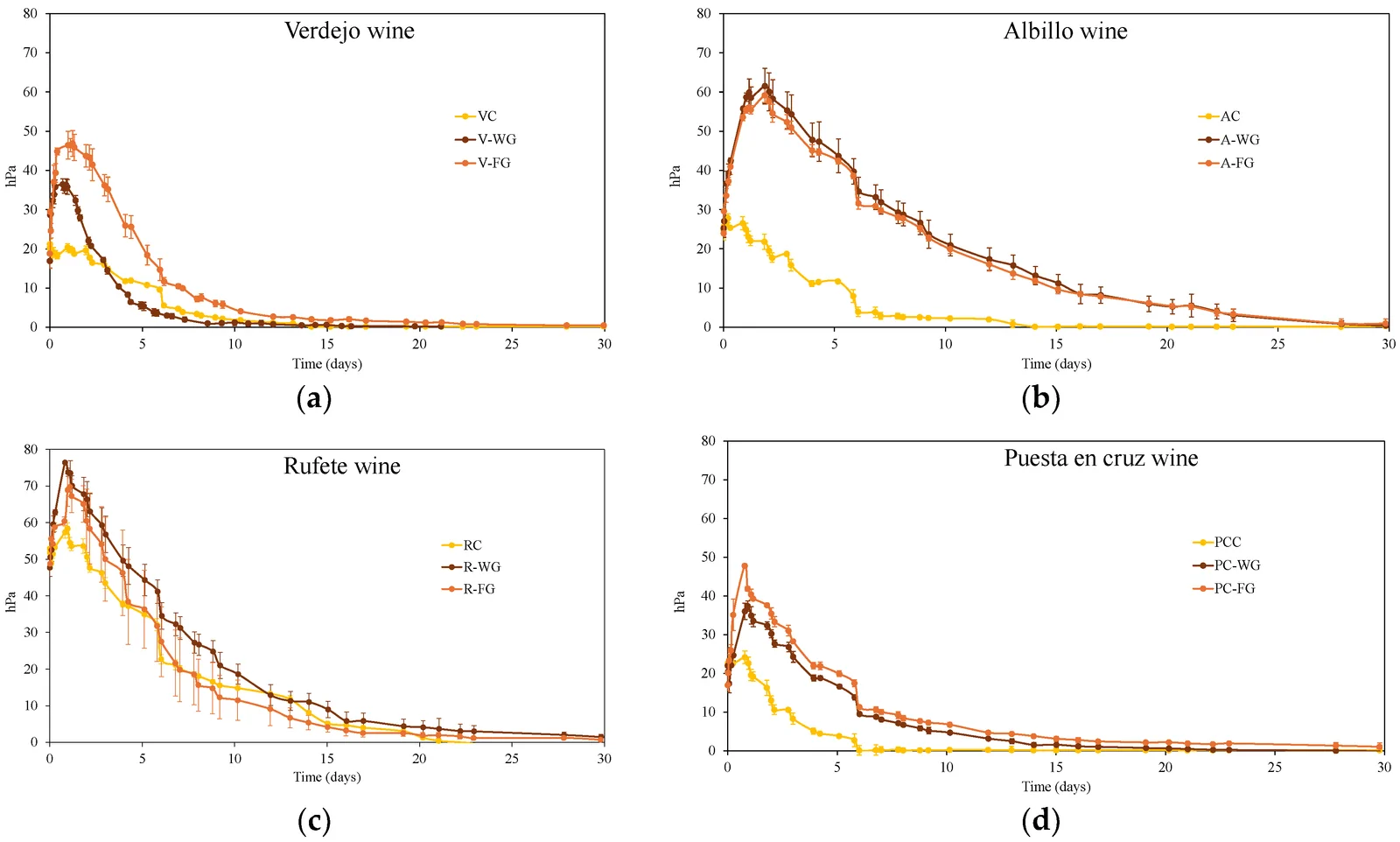
Mean dissolved-oxygen partial-pressure profiles in the monovarietal Verdejo (**a**), Albillo (**b**), Rufete (**c**), and Puesta en Cruz (**d**) wines during the 35-day experiment without wood (control, CT) and in contact with wide-grain (WG) or fine-grain (FG) *Quercus alba* wood. Error bars represent standard deviation (*n* = 2 independent bottles for controls and *n* = 3 for each wood treatment).

Figure 2 shows the evolution of dissolved-oxygen partial pressure during the contact period for the control wines and the wines in contact with WG and FG wood. A similar pattern was observed in the four wines studied: initially, dissolved-oxygen partial pressure increased to a maximum and then progressively decreased to values close to zero. This trend does not reflect oxygen release alone because air release from the wood and oxygen consumption occur simultaneously during the ascending phase, whereas consumption exceeds oxygen supply during the descending phase. The observed profile therefore represents the net oxygen balance between supply from the wood and consumption by compounds already present in the wine or released from the wood. Accordingly, the measured maximum does not represent the total amount of oxygen released by the wood, but rather the oxygen remaining in solution while supply and consumption proceed simultaneously.

Each wine had a different initial dissolved-oxygen partial pressure, reflecting the sum of the oxygen dissolved in the wine and the oxygen incorporated during bottle filling. Accordingly, initial values were close to 20 hPa in Puesta en Cruz and Verdejo, 25 hPa in Albillo, and 50 hPa in Rufete. The initial increase observed in the wood-treated wines is consistent with the self-oxygenation process that occurs as the wood becomes wetted and impregnated with wine. Air present on the surface and, above all, within the pores of the wood is displaced as wine fills these spaces, and part of this oxygen dissolves in the wine. For alternative oak products, this phenomenon has been described as the sum of a rapid release associated with the surface and the initially wetted layers and a slower release linked to the progressive filling of the pore network. Meanwhile, soluble compounds extracted from the wood and the wine’s own constituents consume oxygen. Thus, the dissolved-oxygen profile reflects two opposing, overlapping processes [31].

The Verdejo control reached a maximum close to 20 hPa (Figure 2a), whereas the wood-treated wines reached approximately 35–46 hPa. The FG treatment produced the highest maximum and maintained higher partial pressures throughout much of the experiment. Dissolved oxygen in the control decreased to very low levels after 10 days, whereas it remained detectable for a longer period of time for the wood treatments. Thus, the profile obtained with the fine-grain wood indicated greater net oxygen availability, as reflected by both a higher observed maximum and a longer period during which dissolved oxygen remained measurable, possibly owing to interactions between compounds released by the wood and those already present in the wine.

Albillo showed one of the clearest responses to wood contact (Figure 2b). Both wood treatments reached maxima close to 60 hPa, compared with approximately 25–28 hPa for the control, and the resulting curves nearly overlapped. Beyond increasing the observed maximum, wood contact extended the descending phase of the dissolved-oxygen profile: whereas the measured dissolved-oxygen partial pressure in the control declined to values close to zero in approximately 12 days, the decline was slower in the wines with wood, in which dissolved oxygen remained measurable for more than 28 days, with no difference between the two woods of contrasting ring width.

Rufete wine showed the highest dissolved-oxygen partial pressures of the four varieties studied (Figure 2c), possibly because of its higher initial dissolved-oxygen partial pressure. The control wine began at approximately 55 hPa, whereas the wines with wood reached values close to 70–76 hPa. While the WG treatment produced the highest partial pressures, the FG treatment produced a high maximum but a more rapid subsequent decline. This pattern indicates greater net oxygen availability during the initial phase with FG wood, followed by a more rapid decline in measured dissolved-oxygen partial pressure; the relative contributions of oxygen release and consumption cannot be separated from these profiles.

The measured dissolved-oxygen partial pressure in the control Puesta en Cruz wine declined more rapidly than in the other wines and reached values close to zero within one week. The wines in contact with wood showed a similar pattern: dissolved oxygen increased from 24 hPa to approximately 37 hPa under the WG treatment and 48 hPa under the FG treatment, and remained measurable for a longer period. In these wines, wood contact was associated with a more pronounced increase in net oxygen availability, although the subsequent decline in measured dissolved-oxygen partial pressure remained rapid compared with Albillo and Rufete. Descriptive comparison of the two *Q. alba* wood samples across the four wines revealed no consistent pattern under the experimental conditions tested. The fine-grain wood was associated with the highest observed maxima and greater net oxygen availability in Verdejo and Puesta en Cruz wines; Albillo behaved similarly with both woods, whereas the WG treatment produced higher dissolved-oxygen partial pressures in Rufete. These differences among profiles are consistent with the high variability described for oxygen-consumption kinetics in white wines. The amount of oxygen consumed and its rate of depletion depend on phenolic composition, SO_2_ content, metals, and variety [32]. These comparisons are descriptive and do not imply statistically demonstrated matrix-dependent treatment effects.

In summary, at the onset of wine contact, the wood treatments produced a transient increase in measured dissolved-oxygen partial pressure, consistent with the self-oxygenation process described above, while wood compounds were also being released. Because oxygen consumption occurs simultaneously, these profiles describe net oxygen availability and do not permit oxygen release and consumption to be quantified separately. This behavior cannot be directly extrapolated to the continuous oxygen transfer through barrel staves or to the kinetics of wood-compound release. With alternative products fully submerged in wine, several wood faces are exposed to the wine, and neither continuous micro-oxygenation nor the dynamic oxygen transfer that occurs in a barrel takes place.

As trapped air is displaced and impregnation progresses, the contribution of this initial oxygen supply is expected to diminish, and the net balance shifts toward a progressive decline in measured dissolved-oxygen partial pressure.

### 3.3. General Chemical Composition, Phenolic Indices, and Color After 35 Days

The general chemical attributes of all four varietal wines after 35 days of storage with or without wood contact are presented in Table 3. Monitoring these attributes made it possible to assess their evolution and identify differences among varieties and wood samples. Ethanol content and pH affect the wine’s extraction capacity, whereas SO_2_ participates in the reactions occurring during wine evolution protects some compounds from oxidation. Furthermore, particularly in white wines, its concentration is closely related to oxygen consumption [32,33].

The pH increased in all wines over the 35-day period, although the magnitude depended on the variety. The largest increases were observed in Verdejo and Albillo wines (2%, equivalent to 0.05–0.08 units), whereas Rufete and Puesta en Cruz showed increases of 1% (equivalent to 0.02–0.04 units). The exception was the pH of the PCCT wine, which remained stable. No clear pattern was observed associated with the presence or absence of wood or the wood sample, because each variety followed a different pattern and the differences between controls and wood treatments ranged from 0.00 to 0.04 units. Clear varietal differences were observed: Verdejo had the highest pH, followed by Albillo and Rufete, whereas Puesta en Cruz had the lowest.

Consistent with the increase in pH observed over the study period, total acidity decreased after wood contact in all four varieties. The reduction was minimal in Verdejo (<0.2 g/L), whereas in Albillo, Rufete, and Puesta en Cruz, it ranged from 0.4 to 0.8 g/L. In general, the control wines showed smaller decreases in total acidity than the wines aged with wood (approximately 0.2 g/L less). This pattern was clearest in Albillo, where the wines in contact with wood showed a greater reduction, of approximately 0.3–0.4 g/L.

Two-way ANOVA confirmed that variation in pH and total acidity was driven primarily by wine variety, whose F values were considerably higher than those obtained for wood treatment. Nevertheless, the relative contribution of wood treatment was greater for total acidity than for pH, as reflected by the corresponding F values.

From an enological perspective, the Puesta en Cruz wines, which had the lowest pH and highest total acidity, would be expected to show greater microbiological stability and a fresher sensory profile. However, these characteristics did not translate into a consistently greater relative transfer of wood-derived compounds. In fact, Puesta en Cruz generally showed lower RTI values than the other wine matrices (Section 3.5), indicating that the influence of pH and acidity on compound transfer was compound- and matrix-dependent. In contrast, although the control wines generally maintained slightly higher total acidity than the wood treatments, the observed differences were small and probably of little enological relevance. Correlation analysis showed that ferulic, p-coumaric, chlorogenic, and sinapic acids were positively associated with pH and negatively associated with total acidity. Ferulic, p-coumaric, and sinapic acids were more strongly correlated with pH (r = 0.91, r = 0.41, and r = 0.86, respectively), whereas chlorogenic acid was more closely related to total acidity (r = −0.62).

Volatile acidity in the wines increased by 0.07–0.24 g/L over 35 days. The increase varied mainly among varieties and was greatest in Puesta en Cruz wines, followed by Rufete, Verdejo, and Albillo. In contrast, wood contact did not consistently affect this parameter, because the differences between the control wines and the wood treatments were generally below 0.06 g/L and, in most cases, were not statistically significant. Two-way ANOVA confirmed that variety was the main factor associated with variability in volatile acidity, although wood treatment and its interaction with variety were also significant.

Verdejo and Albillo wines had very similar initial alcohol contents, whereas Rufete showed a slightly higher content (<0.4% *v*/*v*), and Puesta en Cruz had the lowest alcohol content, approximately 0.8% *v*/*v* lower than Rufete. After 35 days of aging, measured alcohol content varied among varieties: Verdejo and Albillo showed no appreciable changes, whereas Rufete and especially Puesta en Cruz decreased. In the two varieties in which changes were observed, the decrease was more pronounced in the wood-treated wines than in the control wines. Among wood-treated wines, the FG treatment produced the greatest reductions in alcohol content (up to 0.5% *v*/*v* in Rufete and 1.0% *v*/*v* in Puesta en Cruz). Two-way ANOVA confirmed a significant effect of both factors on alcohol content (*p* < 0.001), with the influence of variety being considerably greater than that of wood.

Ethanol content is one of the factors that can influence the ability of wine to extract compounds from wood [33]. However, in the present study, alcohol content was associated with few of the phenolic compounds analyzed, showing positive correlations with ferulic and sinapic acids (r = 0.40 and r = 0.45, respectively). The weak association observed between alcohol content and phenolic composition could be explained, at least in part, by the fact that ethanol content and pH did not vary in parallel among varieties, making their individual contributions to the extraction of wood-derived compounds difficult to distinguish. In particular, Puesta en Cruz had both the lowest ethanol content and the lowest pH, making it difficult to isolate their individual contributions to the extraction of wood-derived compounds. This interpretation is consistent with a previous study that found no clear relationship between extraction capacity and ethanol content, possibly because of the narrow range of ethanol concentrations examined [33]. Similarly, the range of variation in alcohol content observed in this study may have limited the detection of associations with phenolic composition.

Both free and total SO_2_ contents decreased in wines in contact with wood, whereas the control wines remained nearly constant during the aging period. Among the wood treatments, the magnitude of this decrease varied among varieties, with the smallest losses of free SO_2_ observed in Puesta en Cruz (<50% relative to the initial value), whereas the remaining varieties showed reductions of more than 50%, with the largest decreases in Rufete (71–77%). Unlike the parameters described above, two-way ANOVA showed that wood contact was the main factor associated with the variability in both free and total SO_2_, with F values higher than those associated with variety. These results indicate that contact with wood was primarily responsible for SO_2_ consumption over the 35-day period, whereas varietal characteristics modulated the magnitude of this decrease. The reduction in SO_2_ content during wood contact is consistent with previous reports and is attributed to SO_2_ consumption in oxidation reactions, as well as to its binding to compounds formed or released during the wine–wood interaction, both of which progressively reduce its concentration [32]. In this context, Puesta en Cruz combined the highest free SO_2_ content with the lowest pH values at the end of wood contact, conditions that favor a higher proportion of molecular SO_2_, the fraction responsible for the antimicrobial and antioxidant activity of wine [34].

**Figure 3:**
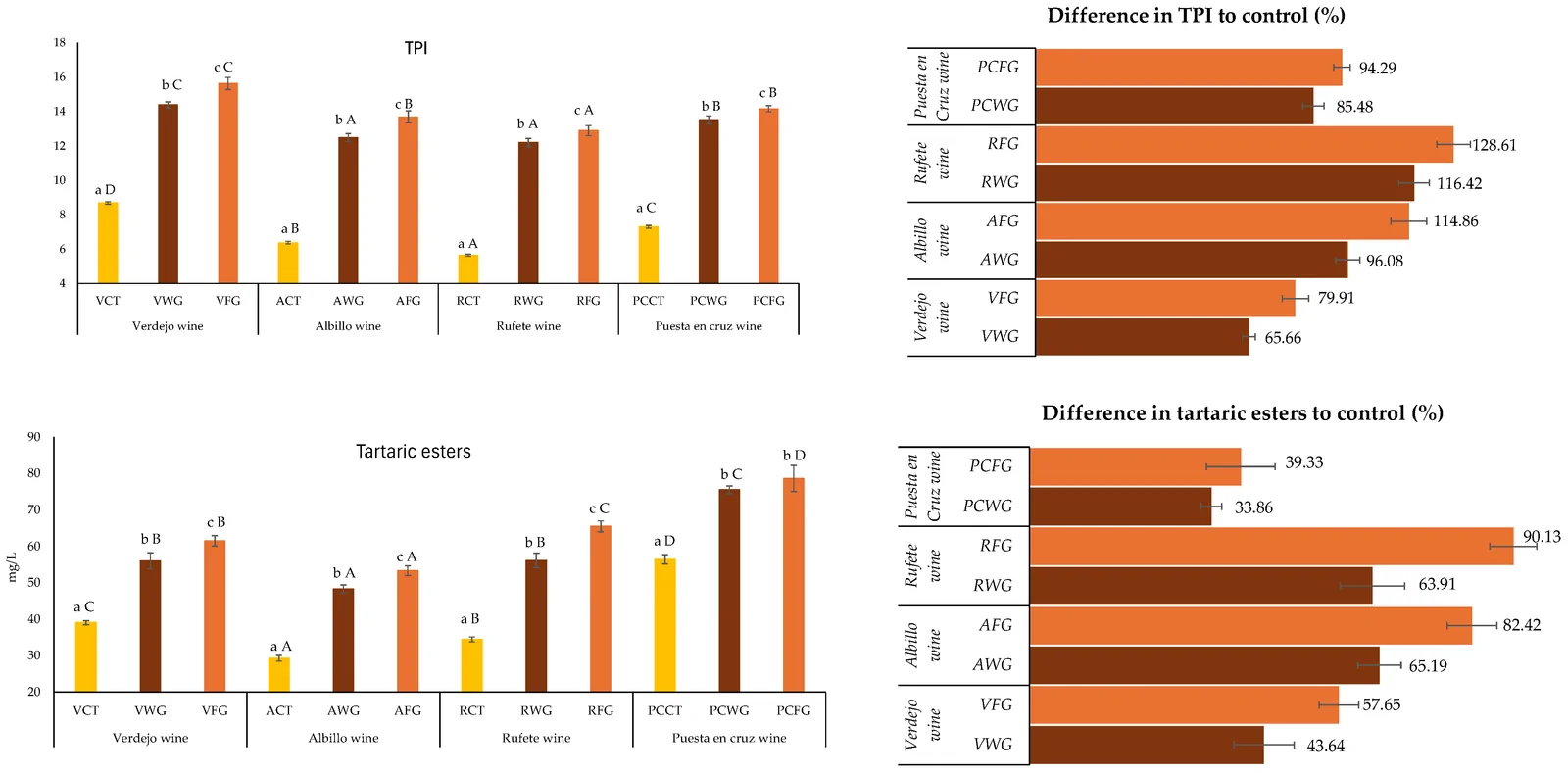

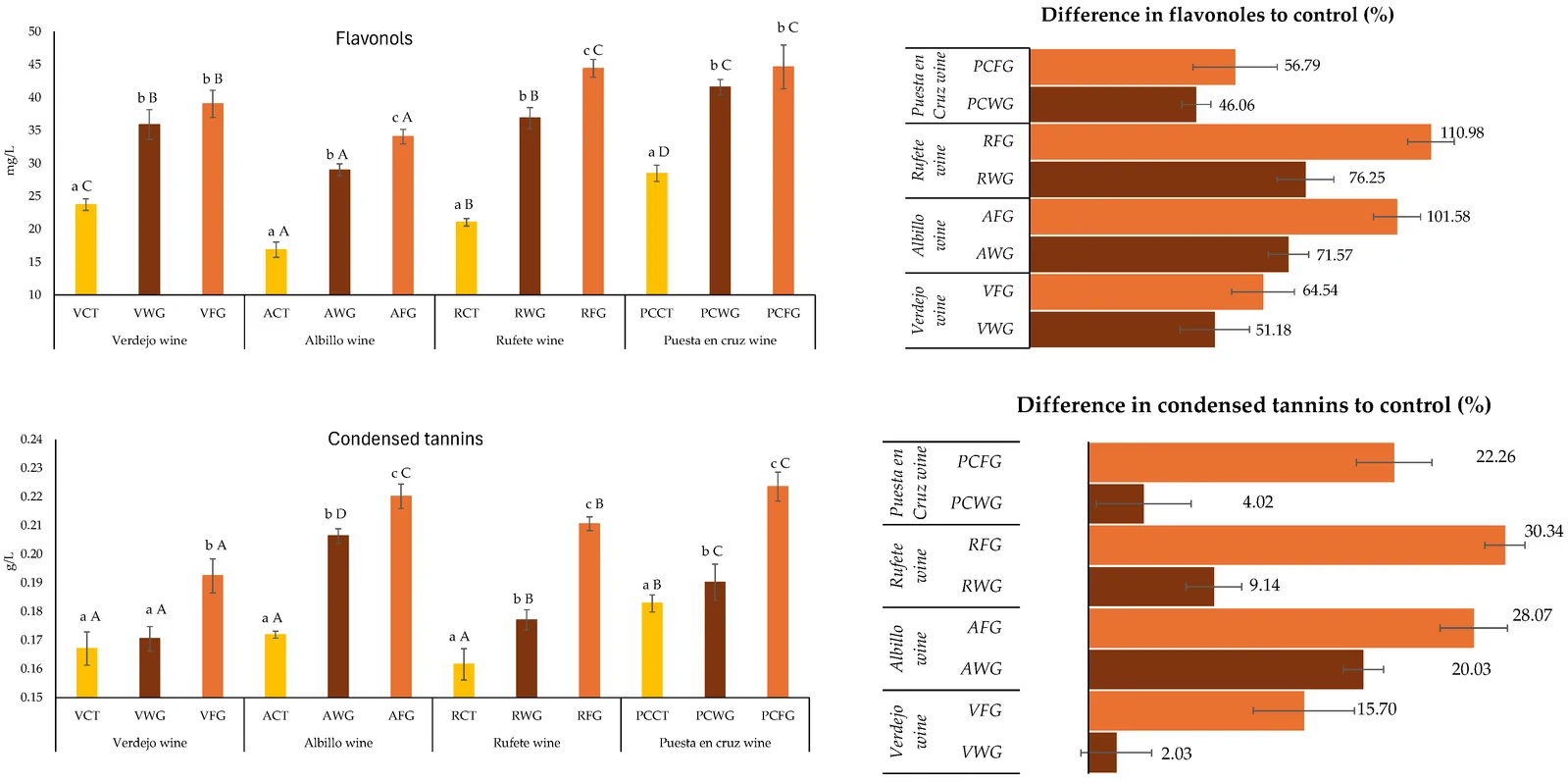
TPI, tartaric esters, flavonols, and condensed tannins in four monovarietal wines (Verdejo, Albillo, Rufete, and Puesta en Cruz) after 35 days, either without wood (control) or with WG or FG wood contact. Data are presented as mean ± standard deviation (SD), with *n* = 2 for control wines and *n* = 3 for wines treated with wood, where *n* represents independent experimental units. Different lowercase letters indicate significant differences among treatments within each wine matrix (a, b, c), whereas different uppercase letters (A, B, C) indicate significant differences among wine matrices within each treatment (Tukey’s HSD test, *p* < 0.05).

Figure 3 presents the concentrations of total phenolic compounds (TPI), tartaric esters, flavonols, and tannins after 35 days of aging. The values obtained for the control wines (CT) and the WG- and FG-treated wines are shown in the left-hand panels, whereas the right-hand panels show the differences between wood-treated wines and their respective controls.

The TPI remained essentially unchanged in the control wines during the study period, whereas it increased in all wines in contact with wood, in agreement with previous reports for this type of treatment [1,6,7,19,35] (Figure 3). The relative increase in TPI tended to be larger in varieties with lower initial phenolic contents; thus, Rufete, which had the lowest initial concentration, showed the largest percentage increase, although its final TPI remained the lowest. Overall, these results suggest that the initial phenolic content of the matrix influenced the magnitude of the increase in TPI during wood contact. In addition, the transfer of phenolic compounds was greater with FG wood than with WG wood. The tartaric ester index also differed among varieties (Figure 3). Puesta en Cruz (PCCT) had the highest concentrations and Albillo (ACT) had the lowest. This pattern agrees with: the concentrations of the main hydroxycinnamic derivatives esterified with tartaric acid, including trans-fertaric and cis-coutaric acids, which were highest in Puesta en Cruz and lowest in Albillo. After 35 days, those wines in contact with wood had higher tartaric ester contents than the control wines. The magnitude of these differences depended on the variety and was more pronounced in Albillo and Rufete, whose controls had lower ester contents, but smaller in Puesta en Cruz, the variety with the highest content of this phenolic fraction. This behavior suggests that the initial composition of the matrix conditioned the response to wood contact, similarly to that observed for the TPI. In addition, wines in contact with FG wood generally had higher tartaric ester contents than WG-treated wines, although this difference was not significant in Puesta en Cruz, consistent with the higher phenolic content of FG wood (Figure 3). The differences observed between wines with and without wood, as well as between the two wood samples, could be related to the differential transfer of phenolic compounds from the wood. The greater availability of these compounds could help preserve tartaric esters during wine evolution, limiting transformations resulting from hydrolysis and oxidation processes.

Flavonols constitute one of the main phenolic fractions of white wines because they contribute to both the yellow hue and antioxidant capacity of wine and play an important role in color evolution [36]. After 35 days of aging, Puesta en Cruz had the highest flavonol concentrations and Albillo had the lowest, mirroring the varietal pattern observed for tartaric esters. In addition, the magnitude of the differences between wines with wood and their respective controls was greater in the varieties with lower contents in the control wines, which, as with the TPI and tartaric esters, suggests that the phenolic composition of the matrix conditioned the response to wood contact. In general, wines treated with FG showed higher flavonol concentrations than those treated with WG, although this difference was not significant in Verdejo and Puesta en Cruz. This trend is consistent with the higher phenolic compound content of FG wood. Moreover, flavonol content showed a very strong positive correlation with absorbance at 420 nm (r = 0.7511; *p* < 0.001), supporting a close relationship between this phenolic fraction and the intensity of the yellow color of white wines.

Tannins constitute one of the main phenolic fractions that contribute to perceived astringency and, to a lesser extent, bitterness and mouthfeel in wines. Although their content in white wines is low, they can contribute to these sensory attributes because of the high acidity of these wines [36]. After 35 days of aging, the wines in contact with wood had higher tannin contents than their respective controls, as previously reported in white wines aged with wood [1]. Unlike the behavior observed for the TPI, tartaric esters, and flavonols, the magnitude of the tannin response was not conditioned by the initial content in the control wines. However, as with the other phenolic fractions, wines treated with FG showed significantly higher contents than those treated with WG. Previous studies have reported higher catechin concentrations in wood-treated white wines than in controls [19]. In the present study, a general trend toward higher catechin and epicatechin contents was also observed in wines in contact with wood. Although wood contact may influence the tannin index of wine, the results obtained suggest that the observed differences may be largely related to greater preservation or stabilization of the wine’s native tannins during wood contact. From an enological perspective, Puesta en Cruz and Albillo wines treated with FG combined the highest tannin contents with the highest acidity levels in the study. Considering that higher acidity and lower ethanol content have been associated with a greater potential perception of astringency and bitterness [37], these wines may therefore be more likely to exhibit these attributes.

Two-way ANOVA confirmed a significant effect of both variety and wood contact on the TPI, tartaric esters, flavonols, and tannins (*p* < 0.001). In all cases, wood contact was associated with larger F values than variety, although the magnitude of this difference depended on the phenolic group considered. This difference was especially marked for the TPI, for which the F value associated with wood was more than thirteen times higher than that associated with variety (4824.79 versus 366.22). Wood contact also yielded higher F values for flavonols (F = 542.17 versus 120.47; approximately 4.5 times higher) and tannins (F = 327.26 versus 68.73; approximately 4.8 times higher). For tartaric esters, although the effect of wood was also greater (F = 1001.31 versus 620.00), the difference between the two factors was considerably smaller (approximately 1.6-fold). The variety × wood interaction was also significant for all four phenolic groups (*p* < 0.001), although its F values were much lower than those of the main effects.

**Figure 4:**
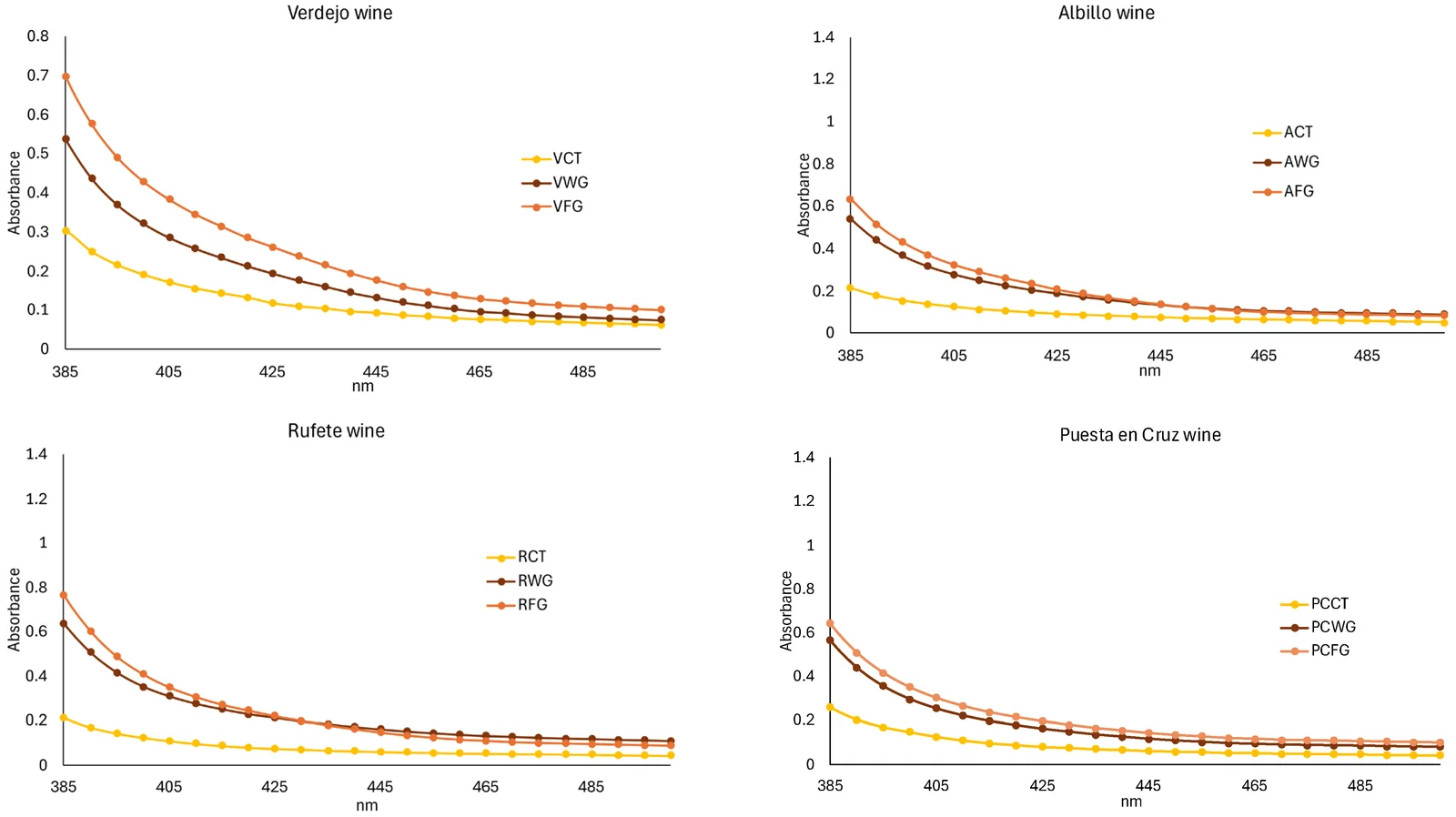

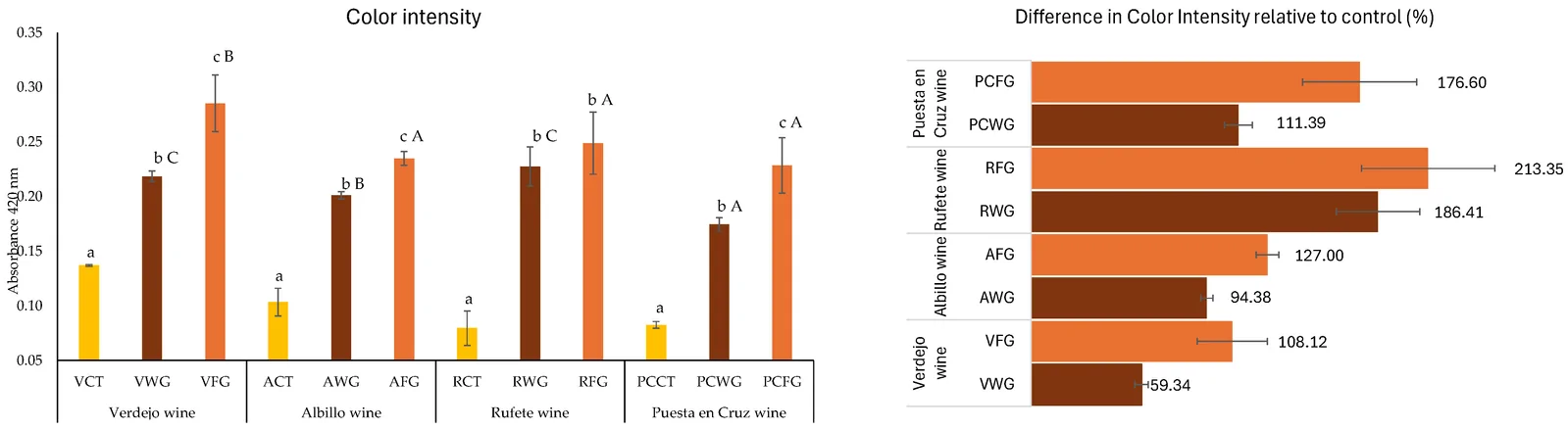
Color intensity and spectrum in four monovarietal wines (Verdejo, Albillo, Rufete, and Puesta en Cruz) after 35 days, either without wood (control) or with WG or FG wood contact. Data are presented as mean ± standard deviation (SD), with *n* = 2 for control wines and *n* = 3 for wines treated with wood, where *n* represents independent experimental units. Different lowercase letters indicate significant differences among treatments within each wine matrix (a, b, c), whereas different uppercase letters (A, B, C) indicate significant differences among wine matrices within each treatment (Tukey’s HSD test, *p* < 0.05).

Figure 4 shows color intensity (CI), the percentage difference in CI relative to each control, and the absorbance spectra after 35 days. Wines in contact with wood showed increased CI, consistent with previous studies [7,32,38]. This increase likely reflects reactions between wood-derived compounds and wine constituents that alter wine color. In contrast, CI remained essentially unchanged in all control wines without wood contact. A significantly greater increase was observed in all wines in contact with FG wood than in those with WG wood, except for the Rufete wines, for which the increases were similar for both woods and were the largest relative to the control.

### 3.4. Low-Molecular-Weight Phenolic Compounds in Wood Samples and Wood-Treated Wines

**Figure 5:**
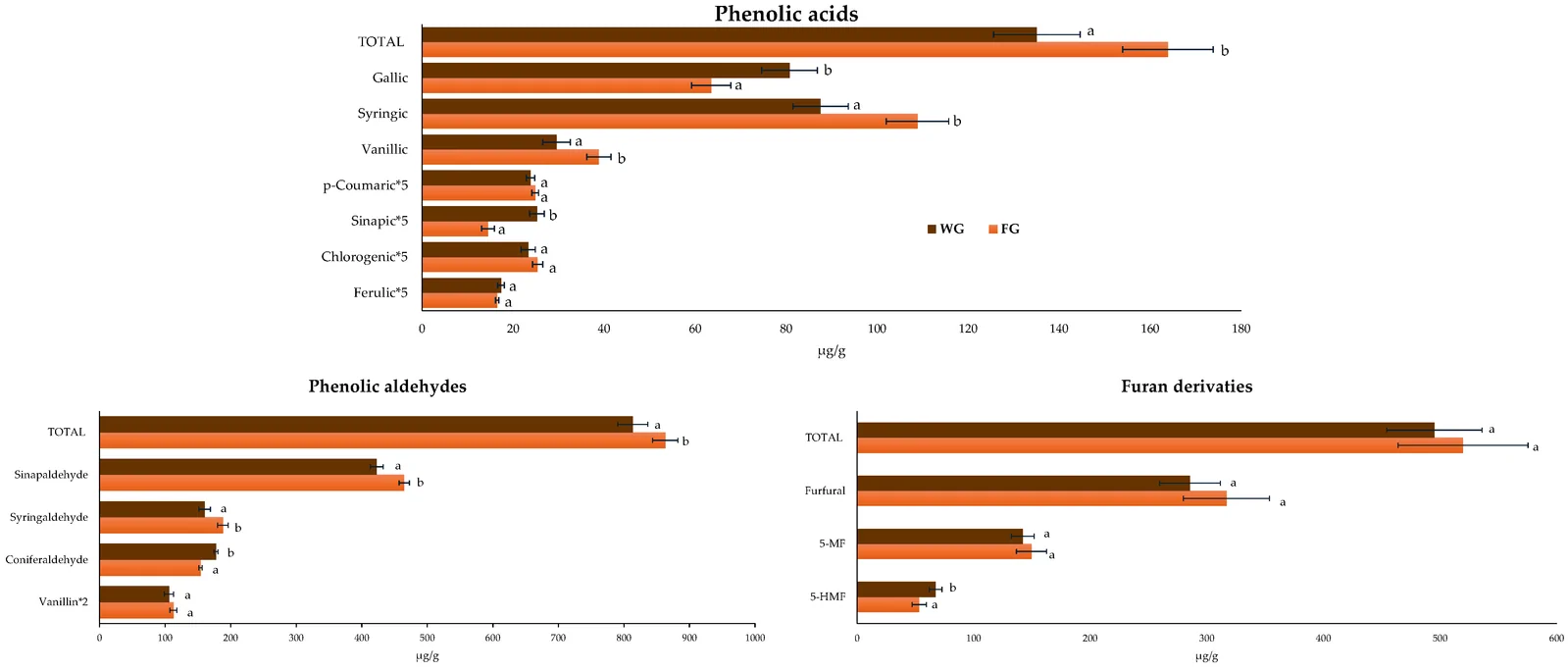
Concentration of phenolic acids (gallic, syringic, vanillic, p-coumaric, sinapic, chlorogenic, and ferulic acids), phenolic aldehydes (sinapaldehyde, syringaldehyde, coniferaldehyde, and vanillin), and furanic compounds (furfural, 5-methylfurfural, and 5-hydroxymethylfurfural) in WG and FG wood extracts (µg/g of wood). Error bars represent standard deviation. Different lowercase letters indicate significant differences between WG and FG wood (a, b). *5 = values multiplied by 5; *2 = values multiplied by 2. Total concentrations represent the sum of the individual compounds included in each phenolic compound family.

**Figure 6:**
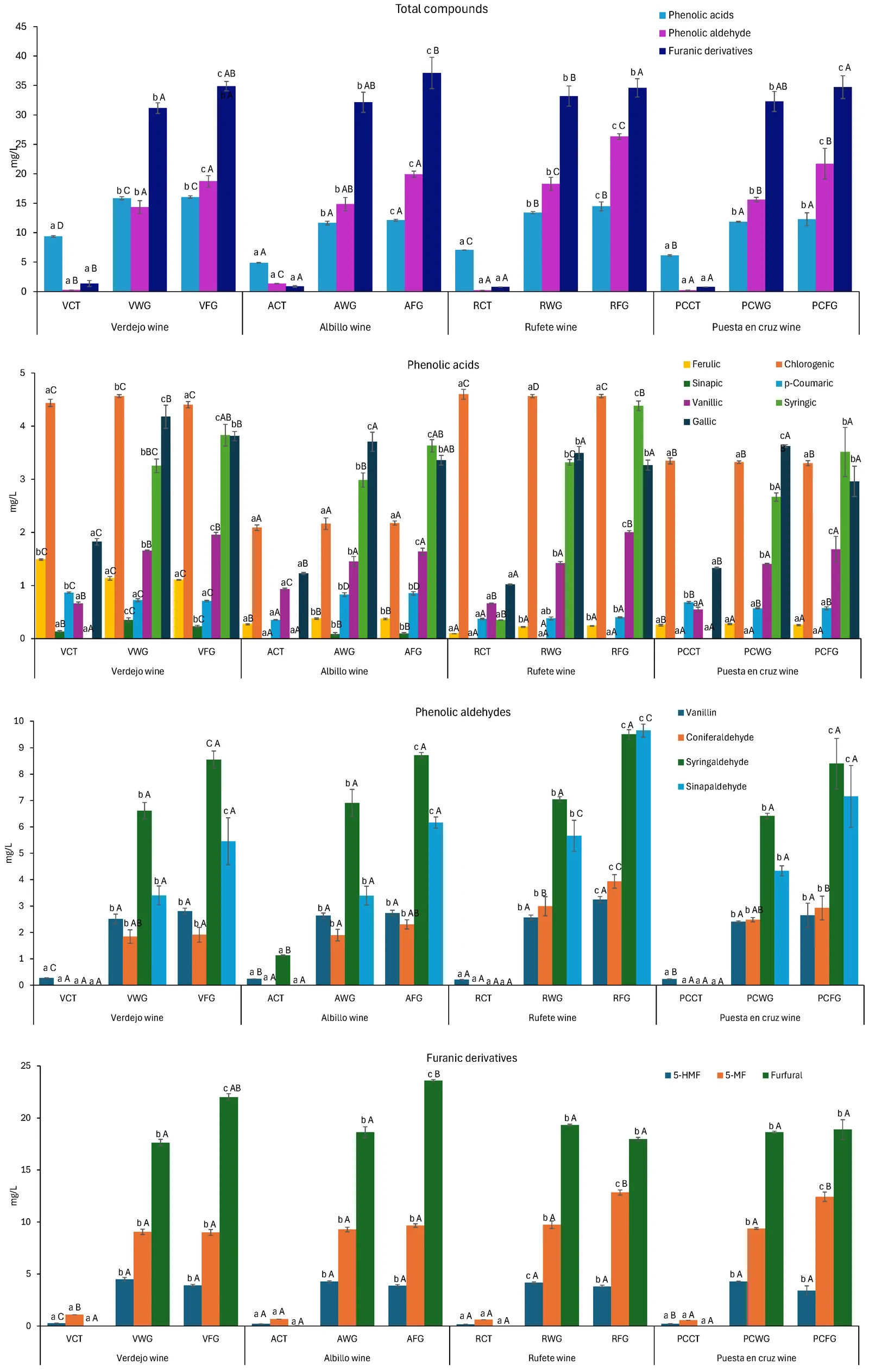
Concentration of phenolic acids (gallic, syringic, vanillic, p-coumaric, sinapic, chlorogenic, and ferulic acids), phenolic aldehydes (sinapaldehyde, syringaldehyde, coniferaldehyde, and vanillin), and furanic compounds (furfural, 5-methylfurfural, and 5-hydroxymethylfurfural) in four monovarietal wines (Verdejo, Albillo, Rufete, and Puesta en Cruz) after 35 days, either without wood (control) or with WG or FG wood contact. Data are presented as mean ± standard deviation (SD), with *n* = 2 for control wines and *n* = 3 for wines treated with wood, where *n* represents independent experimental units. Total concentrations represent the sum of the individual compounds included in each phenolic compound family. Different lowercase letters indicate significant differences among treatments within each wine matrix (a, b, c), whereas different uppercase letters (A, B, C) indicate significant differences among wine matrices within each treatment (Tukey’s HSD test, *p* < 0.05).

Figure 5 and Figure 6 present the phenolic acids, phenolic aldehydes, and furanic derivatives identified and quantified in the wood samples and the corresponding wines after 35 days of aging, respectively. Table 4 summarizes the results of the two-way ANOVA for the compounds quantified in the wines, showing the effects of wine matrix, wood treatment, and their interaction. In addition, Table 5 presents the compounds quantified in the wines but not detected in the wood samples.

Syringic acid was the predominant phenolic acid in both wood samples, followed by gallic acid. Although gallic acid is usually considered one of the main phenolic acids in oak wood, second only to ellagic acid [39], higher concentrations of syringic acid have previously been reported in *Quercus alba* [40,41]. Published differences indicate that the phenolic composition of wood can vary according to species and the toasting conditions used, among other factors [39]. The two wood samples differed: FG wood had significantly higher concentrations of syringic acid (108.9 µg/g) than WG wood (87.6 µg/g), whereas gallic acid showed the opposite pattern, with higher concentrations in WG wood (80.8 versus 63.6 µg/g). Vanillic acid was the third most abundant compound in both woods, with significantly higher concentrations in FG wood (38.8 µg/g) than in WG wood (29.6 µg/g). The remaining compounds (p-coumaric, sinapic, ferulic, and chlorogenic acids) were present at considerably lower concentrations, generally below 5 µg/g. Of these, p-coumaric, ferulic, and chlorogenic acids showed no significant differences between the two woods, whereas sinapic acid was more abundant in WG wood.

Among the compounds identified in both wood and wine, phenolic acids were the predominant family in the control wines. However, after contact with wood, furanic derivatives became the predominant family, generally followed by phenolic aldehydes. The only exception was the Verdejo-WG wine, in which the contents of phenolic acids and phenolic aldehydes were similar (Figure 6).

Among the phenolic acids identified in both wood and wine, chlorogenic acid was the predominant compound in the wines and varied markedly among varieties (Table 4), with the highest concentrations in Verdejo and Rufete and the lowest in Albillo (Figure 6). However, its concentration changed very little after wood contact, remaining similar to that observed in the respective control wines (Figure 6). This behavior is consistent with the low concentrations of chlorogenic acid determined in both woods. After chlorogenic acid, gallic acid was the most abundant phenolic acid in the control wines, whereas the third most abundant phenolic acid depended on the variety. However, the profile of the main phenolic acids in the wines after wood contact reflected the composition of the wood. Thus, in wines treated with FG wood, syringic acid generally reached concentrations higher than those of gallic acid, whereas in wines treated with WG wood, gallic acid remained the second most abundant phenolic acid, with vanillic acid ranking as the fourth most abundant compound.

Wood contact significantly affected the concentrations of gallic, syringic, and vanillic acids (Figure 6), the three predominant phenolic acids identified in the woods (Figure 5). In all three cases, the effect of wood contact was greater than that of variety (Table 4), indicating that wood composition was the main factor influencing their final concentration in the wines. Nevertheless, variety also had a significant effect, demonstrating that matrix composition influenced the magnitude of the response to wood contact. Thus, the higher gallic acid content of WG wood and the higher syringic and vanillic acid contents of FG wood were reflected in the wines made with each wood, supporting direct transfer from the wood. In addition, the magnitude of the increase depended on variety: increases were smaller in varieties with higher control concentrations and larger in those with the lowest, indicating that matrix composition also modulated the incorporation of these compounds during wood contact. Although wood contact increased the concentrations of gallic, syringic, and vanillic acids, their concentrations in the wines were below their respective perception thresholds [42]. Therefore, based on published sensory thresholds, these compounds would be expected to make only a limited direct contribution to body, viscosity, unctuousness, bitterness, astringency, or acidity. Nevertheless, their presence may contribute to the antioxidant properties of the wine and could still influence sensory properties [43].

Sinapic, ferulic, and p-coumaric acids were found at low concentrations in both wood samples and the wines. Sinapic acid was detected only in the Verdejo control wine and increased after wood contact, particularly after WG treatment, in agreement with the composition of this wood. Nevertheless, this behavior depended on variety, because no differences between woods were observed in Albillo, and sinapic acid was not detected in Rufete or Puesta en Cruz (Figure 6). Ferulic acid was found at concentrations below 0.4 mg/L in most wines, except Verdejo, which showed higher concentrations of approximately 1.1 mg/L (Figure 6). Unlike gallic, syringic, and vanillic acids, wood contact did not have a uniform effect on this compound: its content decreased in Verdejo wines, remained unchanged in Puesta en Cruz wines, and increased to a similar extent with both woods in Albillo and Rufete wines. Similar behavior was observed for p-coumaric acid, whose concentration decreased in the varieties with higher contents in the control wines (Verdejo and Puesta en Cruz), remained practically constant in Rufete, and increased in Albillo. Consequently, two-way ANOVA showed that, for both ferulic and p-coumaric acids, variety produced larger F values than wood contact, and the variety × wood-treatment interaction also produced larger F values than wood treatment (Table 4). These results are consistent with the low concentrations of both compounds determined in the woods, suggesting that the contribution of wood to their final concentrations in the wines was limited under the conditions tested.

Aldehydes undergo the largest increases during wood toasting [39]. Accordingly, phenolic aldehydes were the predominant family of compounds in both WG and FG woods, with concentrations above 800 µg/g, far higher than those determined for furanic derivatives (495–520 µg/g) and phenolic acids (135–164 µg/g) (Figure 5). Among the phenolic aldehydes, sinapaldehyde was the predominant compound, followed by coniferaldehyde or syringaldehyde, depending on the wood sample, whereas vanillin showed the lowest concentrations. Previous studies on *Quercus alba* have likewise reported marked variation in the relative abundance of these compounds. Some studies identify coniferaldehyde as the predominant phenolic aldehyde [40], whereas others identify sinapaldehyde as the predominant compound [39,41]. Across these studies, vanillin is present at lower concentrations than the other phenolic aldehydes, indicating that the profile of these compounds depends on both the initial properties of the wood and the toasting conditions. In the present study, FG wood had higher concentrations of phenolic aldehydes than WG wood, mainly because of its higher concentrations of sinapaldehyde and syringaldehyde, whereas coniferaldehyde was more abundant in WG wood. In contrast, vanillin concentrations were similar in both woods.

In the control wines, only vanillin (at concentrations below 0.3 mg/L) and, in Albillo, syringaldehyde (1.1 mg/L) were detected (Figure 6). However, after wood contact, the total phenolic aldehyde content reached 14.4 to 26.3 mg/L, consistent with substantial transfer of these compounds from wood to wine. Accordingly, two-way ANOVA showed that the F values for wood contact were much larger than those for variety for all aldehydes (Table 4). Nevertheless, variety also had a significant effect, indicating that matrix composition influenced the final concentrations of these compounds during wood contact. Thus, Rufete wines generally showed the largest increases, especially after contact with FG wood.

Although sinapaldehyde was the predominant phenolic aldehyde in the woods, syringaldehyde predominated in most wines after wood contact, except in Rufete treated with FG wood. In addition, wines treated with FG wood had the highest concentrations of sinapaldehyde and syringaldehyde, in agreement with the higher concentrations of both compounds in FG wood. Vanillin concentrations were similar in both woods. However, no significant differences between FG and WG were observed in Verdejo, Albillo, or Puesta en Cruz wines, whereas Rufete wines in contact with FG wood contained significantly more vanillin than the corresponding WG-treated wines, suggesting that the wine matrix influenced the final concentration of this compound. In contrast, coniferaldehyde showed a different behavior. Although WG wood had higher concentrations of this compound, wines treated with FG wood generally showed higher concentrations; however, the difference was not significant in Albillo and Puesta en Cruz, suggesting that the final concentration of coniferaldehyde was influenced not only by wood composition but also by the wine matrix. Vanillin is considered one of the phenolic aldehydes of greatest sensory relevance in wood-aged wines and is associated with vanilla-like sensory notes [44]. In this study, all wood-treated wines contained vanillin above its reported perception threshold (1000 µg/L), suggesting that vanillin may have been sensory-relevant under the conditions studied [44]. In contrast, syringaldehyde concentrations remained below its reported perception threshold (50 mg/L), whereas sufficiently established sensory thresholds are not available for coniferaldehyde and sinapaldehyde to reliably assess their potential individual sensory contributions [44].

Furanic derivatives constituted the second most abundant family of compounds in the wood samples analyzed (Figure 5). Their formation is associated with the thermal degradation of polysaccharides during wood toasting [39]. Among them, furfural was the predominant furanic derivative (Figure 5), in agreement with previous reports for *Quercus alba* wood [39]. In contrast, the concentrations of 5-methylfurfural (5-MF) and 5-hydroxymethylfurfural (5-HMF) vary considerably among studies because of both the inherent heterogeneity of the species and especially the toasting conditions applied [39]. In the present study, 5-MF was also more abundant than 5-HMF in both woods (Figure 5). The ratio of the two compounds also varies markedly among studies of *Quercus alba*, reflecting both species heterogeneity and toasting conditions [39]. In the wood samples analyzed, no significant differences were observed between the two wood samples for any of the furanic derivatives.

Furfural was not detected in the control wines, whereas 5-hydroxymethylfurfural (5-HMF) and 5-methylfurfural (5-MF) were found only at low concentrations, resulting in total furanic derivative contents ranging from 0.77 to 1.38 mg/L (Figure 6). However, wood contact resulted in the presence of all three compounds, increasing the total furanic derivative content to values between 31.2 and 37.1 mg/L. As observed for phenolic aldehydes, these results illustrate the marked effect of wood contact on the concentration of furanic derivatives in the wines. In addition, furanic derivatives followed the same order of abundance in the wines as in the woods, with furfural as the predominant compound, followed by 5-MF and 5-HMF. Consistent with the higher 5-HMF concentration measured in WG wood, wines in contact with wood showed higher concentrations of this compound than the controls, although the difference between WG and FG was significant only in Rufete wines, where the WG treatment produced the highest concentration (Figure 6). However, the behavior of furfural and 5-MF depended on variety. Thus, furfural did not differ between woods in Rufete and Puesta en Cruz, whereas Verdejo and Albillo wines treated with FG wood reached the highest concentrations. Similarly, 5-MF showed no differences between woods in Verdejo and Albillo, whereas in Rufete and Puesta en Cruz, the highest concentrations occurred with FG wood. Overall, these results, together with the two-way ANOVA, indicate that wood treatment was the main factor determining the concentration of furanic derivatives, although variety also had a significant effect. Thus, higher furfural concentrations were observed in Verdejo and Albillo wines after contact with FG wood; the highest 5-MF concentrations were observed in Rufete and Puesta en Cruz wines with FG wood; and the numerically highest 5-HMF concentration was observed in Verdejo wines treated with WG wood. Furanic derivatives are associated with bread, almond, caramel, and toasted-sugar aromas and can contribute to the aromatic complexity of wines aged in wood [44]. In this study, only furfural exceeded its reported perception threshold (15 mg/L) in wood-treated wines, indicating that it may have been the most sensory-relevant among the furanic derivatives analyzed [44]. The concentrations of 5-methylfurfural (16 mg/L) and 5-hydroxymethylfurfural (100 mg/L) remained below their respective reported perception thresholds [44].

Unlike the compounds discussed above, the compounds listed in Table 5 were detected only in the wines and not in the wood samples analyzed. Their concentrations were driven mainly by variety, which yielded much larger F values than wood treatment or the interaction. Catechin occurred at the highest concentrations (Table 5). Catechin concentrations were generally higher in wines in contact with wood than in their respective controls, except for Puesta en Cruz. In Verdejo, both wood treatments showed higher concentrations than the control, whereas in Rufete the highest concentration was observed with WG wood. Cis-coutaric acid followed a similar pattern, with higher concentrations in wines in contact with wood for all varieties except Verdejo wines. However, epicatechin and protocatechuic, trans-fertaric, and p-coumaric acids were the only compounds for which wood contact did not show a significant effect, with concentrations generally remaining similar to those of the respective control wines. Nevertheless, protocatechuic acid showed a slight increase in Puesta en Cruz, whereas trans-fertaric acid concentrations remained similar among treatments in Verdejo and Albillo. In Puesta en Cruz, the control wine had a higher concentration than both wood-treated wines. In contrast, caffeic acid and tyrosol tended to decrease after wood contact, although this response was not consistent across all varieties. Differences between WG and FG wood were generally limited and depended on the compound and wine variety. The increases observed for catechin, cis-coutaric acid, or, to a lesser extent, trans-fertaric acid may reflect enhanced stability against degradation due to the incorporation of wood-derived phenolic compounds with antioxidant capacity. In contrast, the decreases observed for caffeic acid and tyrosol may be related to adsorption onto the wood or to chemical reactions occurring during wood contact.

### 3.5. Relative Transfer Index of Compounds from Wood to Wine

The relative transfer index (RTI) was evaluated by comparing the concentrations measured in the wines after 35 days of wood contact with those obtained in the corresponding model wine under the same experimental conditions. This approach provides a relative measure of the transfer of each compound from wood to wine and does not represent the total amount extractable from the wood. This distinction is relevant when determining how long alternative oak products can be used or reused. The experiment evaluated wood pieces fully submerged in wine, a wine–wood interaction that differs markedly from barrel aging. When alternative products are fully submerged, wine contacts the radial, tangential, and transverse faces of the wood. Exposure of all three faces may facilitate extraction from wood structures that are less accessible in barrels; it may also alter the profile of compounds released and reduce the influence of cutting orientation.

Most RTI values ranged from 20% to 100% and varied according to the chemical family of the compound: furanic aldehydes (furfural, 5-HMF, 5-MF) and lignin-derived benzoic aldehydes (vanillin, syringaldehyde) showed the highest RTI values, whereas cinnamic aldehydes (coniferaldehyde, sinapaldehyde) showed the lowest (Table 6).

Differences in wood structure may affect transfer kinetics and the extent of transfer through the effective porosity of each wood structure (earlywood, latewood, and medullary rays). In the literature, wood-piece size has repeatedly been identified as an important determinant of wine phenolic composition, sometimes outweighing seasoning or toasting because it determines the surface area available for compound diffusion into the wine [45]. In this study, however, the WG and FG pieces were the same size, so the observed differences may instead be related to density and the anatomical characteristics associated with grain width.

For benzoic and furanic aldehydes, RTI values for FG and WG wood were very similar in most wines. For example, the syringaldehyde RTI in Verdejo was 87.1% with WG wood and 85.4% with FG wood, whereas the vanillin RTI in Puesta en Cruz was 78.8% and 73.6%, respectively. These results indicate that, under the conditions tested, both wood samples showed similar relative transfer of these compounds.

The most marked difference between the two woods was found for cinnamic aldehydes and furanic derivatives in Rufete wine, where RTI values obtained with FG wood exceeded those obtained with WG wood (50.0% versus 38.5% for coniferaldehyde, 46.0% versus 35.4% for sinapaldehyde, and 105.4% versus 88.2% for 5-MF). These differences may be related to the lower density and greater effective porosity of FG wood, which may have favored the transfer of these compounds under the conditions tested.

Differences in RTI among wines were associated mainly with their physicochemical composition, particularly pH, acidity, alcohol content, and total polyphenol index (TPI). Rufete wine, with a low pH (3.21) and high alcohol content (13.74%), showed the highest RTI values for wood-derived compounds, especially cinnamic and furanic aldehydes.

pH and acidity affect the stability of furanic compounds and the condensation reactions of phenolic derivatives. Wines with lower acidity (Rufete and Verdejo) yielded RTIs for furfural, 5-HMF, and 5-MF close to or above 100% relative to the model wine. RTI values above 100% observed for 5-HMF, furfural, and 5-MF in several wines with FG wood (e.g., furfural in Albillo, 112.9%, or 5-HMF in Albillo, 101.5%) mean that net concentrations in the real wine exceeded those in the model wine. This may reflect matrix-dependent differences in compound formation or degradation associated with pH and composition.

Puesta en Cruz wine, with high total acidity (7.6 g/L) and a very low pH (3.05), generally showed the lowest overall RTI values (total acids between 56% and 59%), suggesting that net transfer of wood-derived compounds was lower in this matrix or that some extracted compounds reacted with native wine constituents. The specific and sometimes nonlinear influence of the matrix on the extraction of individual compounds has been described for wines treated with alternative wood products, for which responses are not always uniform across phenolic compounds [46].

The benzoic aldehydes from wood (vanillin, syringaldehyde) showed consistently high RTI values (mostly 74–96%) in all wines and with both WG and FG wood. In Rufete, which had the highest alcohol content, the RTI reached 96.0% for syringaldehyde and 91.7% for vanillin. These lignin-degradation products showed similar relative transfer from the two woods under the conditions tested. For cinnamic aldehydes (coniferaldehyde, sinapaldehyde), RTI values were lower (21–50%), reflecting their lower release into real wine relative to the model.

Phenolic acids (gallic, vanillic, and syringic acids) showed intermediate-to-high RTI values (mostly 45–93%), with the greatest transfer observed for syringic acid (76–93%), whereas vanillic acid was the most variable (30–62%) and highly sensitive to the wine matrix.

Finally, furanic compounds (furfural, 5-HMF, and 5-MF) yielded RTI values close to or above 100% (mostly 76–113%), particularly for FG wood. Because these compounds are concentrated in the toasted surface layer, their RTI may reflect both their distribution within the wood and their matrix-dependent stability and transformation, which are influenced by factors such as pH.

### 3.6. Classification of the Wines

**Figure 7:**
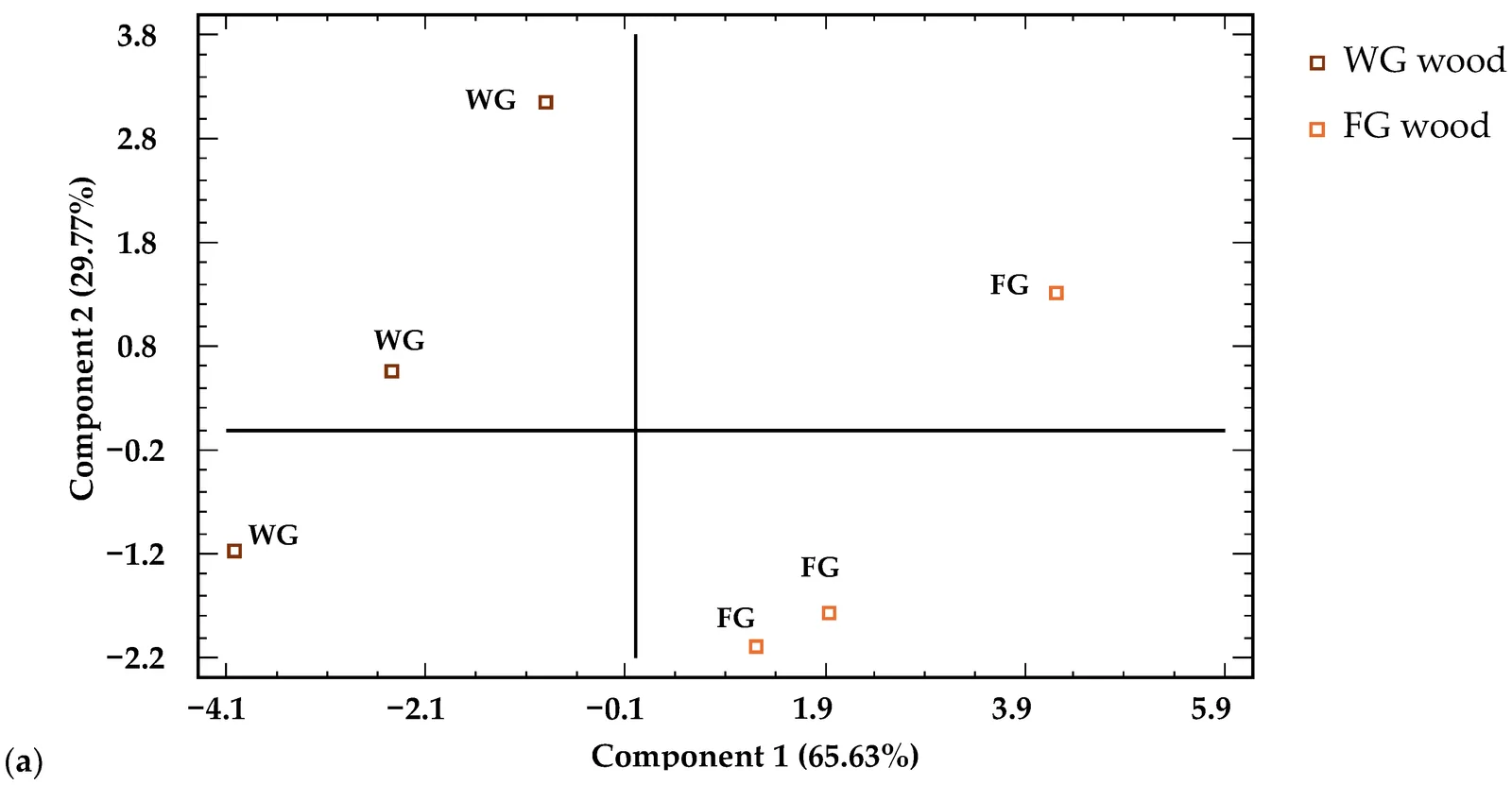

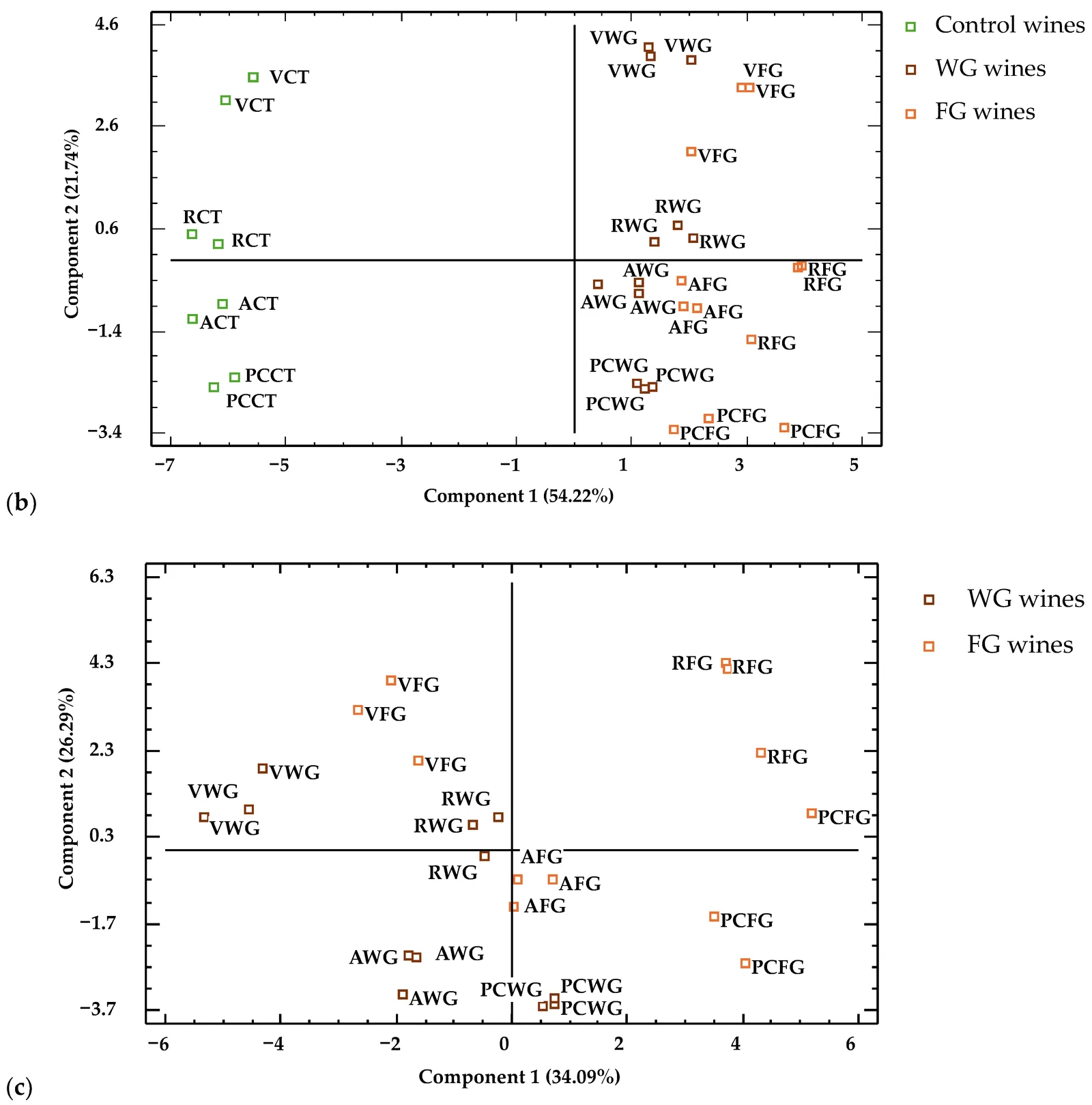
PCA plots for the first two principal components: (**a**) wood samples; (**b**) all wines (controls and wines in contact with wood); (**c**) only wines in contact with WG and FG wood. Each point represents one independent experimental unit.

Principal component analysis (PCA) was used to visualize differences among the wood samples and wines based on their phenolic composition and to identify the variables associated with sample separation. Figure 7 shows the three PCA models obtained: (a) the wood samples, (b) all wines (controls and wines in contact with wood), and (c) only the wines in contact with WG and FG wood.

The PCA of the wood samples (Figure 7a) accounted for 95.41% of the total variability, with the first (PC1) and second (PC2) components accounting for 65.63% and 29.77%, respectively. Samples separated mainly along PC1, with the WG samples scoring negatively and the FG samples positively. This separation reflected differences in the phenolic composition of the two woods. Thus, WG wood was associated with higher contents of coniferaldehyde, sinapic acid, gallic acid, 5-hydroxymethylfurfural, and ferulic acid, whereas FG wood was associated with higher concentrations of most phenolic compounds, particularly vanillic acid, syringic acid, syringaldehyde, and sinapaldehyde. This pattern is consistent with the phenolic profiles of the two wood samples described above. The PCA of all wines, with and without wood contact (Figure 7b), accounted for 75.95% of the total variability, with PC1 and PC2 accounting for 54.22% and 21.74%, respectively. PC1 distinguished the control wines from those in contact with wood, with the controls located on the negative side and the wood-treated wines on the positive side. This separation was mainly associated with higher total polyphenol index (TPI), color intensity (CI), tartaric esters, flavonols, tannins, and concentrations of most wood-derived phenolic compounds, especially vanillic, syringic, and gallic acids; the phenolic aldehydes (vanillin, coniferaldehyde, syringaldehyde, and sinapaldehyde); and the furanic derivatives (furfural, 5-HMF, and 5-MF) (Table 7). PC2 primarily separated Verdejo and Puesta en Cruz wines, whereas Albillo and Rufete occupied intermediate positions, with Verdejo wines on the positive side and Puesta en Cruz wines on the negative side. Positive PC2 scores were associated with alcohol content, pH, and ferulic and sinapic acids, whereas negative scores were associated with total acidity, volatile acidity, tannins, and, to a lesser extent, tartaric esters.

The PCA restricted to the wines in contact with wood (Figure 7c) accounted for 60.38% of the total variability, with PC1 and PC2 accounting for 34.09% and 26.29%, respectively. PC1 consistently tended to separate the WG and FG treatments within each variety, with wines treated with WG wood scoring more negatively than their corresponding FG wines. Nevertheless, this separation was less marked than that observed between the control wines and the wines in contact with wood (Figure 7b), suggesting that wood contact produced greater differences than those observed between the two woods. This trend was associated with higher contents of gallic acid, sinapic acid, ferulic acid, and 5-hydroxymethylfurfural in wines treated with WG, whereas the corresponding FG wines were associated with higher concentrations of 5-methylfurfural, coniferaldehyde, sinapaldehyde, syringaldehyde, vanillin, and, to a lesser extent, vanillic and syringic acids.

## 4. Conclusions

Under the conditions of this study, 35 days of contact with toasted *Quercus alba* pieces modified both net dissolved-oxygen dynamics and the phenolic composition of the four white wines analyzed, reflecting the combined effects of oxygen release and consumption by wine constituents and wood-derived compounds. The balance between release and consumption depended on the wine matrix, with no uniform response to the two woods of contrasting ring width across all wines. FG wood had a lower density and greater impregnation and generally produced larger increases in the total polyphenol index, absorbance at 420 nm, and the phenolic indices measured. Differences in the composition of the two woods were also reflected in the wines: the FG wood was mainly associated with higher concentrations of syringic and vanillic acids, syringaldehyde, sinapaldehyde, and some furanic derivatives, whereas WG wood was associated with higher contents of gallic acid, sinapic acid, and 5-hydroxymethylfurfural. Nevertheless, the final concentration of each compound depended on the initial wine composition, indicating a strong matrix effect. Phenolic aldehydes and furanic derivatives yielded the highest RTI values, whereas phenolic acids showed more variable RTI values. Overall, the results indicate that the selection of alternative *Quercus alba* products should consider not only ring width but also wood composition and the characteristics of the target wine. These findings are limited to two specific *Quercus alba* wood samples tested as fully submerged pieces. Therefore, the observed differences cannot be attributed solely to ring width or generalized to other *Q. alba* wood samples or to the continuous oxygen transfer occurring during barrel aging.

## Figures and Tables

**Table 1:** General chemical characteristics of monovarietal Verdejo, Albillo, Rufete, and Puesta en Cruz wines used in the study.

Parameters	Verdejo	Albillo	Rufete	Puesta en Cruz
pH	3.55 ± 0.01	3.19 ± 0.01	3.21 ± 0.01	3.05 ± 0.01
Total acidity (g/L)	4.0 ± 0.1	6.9 ± 0.0	5.8 ± 0.1	7.6 ± 0.0
Volatile acidity (g/L)	0.22 ± 0.00	0.56 ± 0.11	0.32 ± 0.03	0.16 ± 0.02
Alcohol content (% *v*/*v*)	13.40 ± 0.14	13.30 ± 0.28	13.74 ± 0.27	12.90 ± 0.00
F-SO_2_ (mg/L)	30 ± 0	33 ± 1	31 ± 1	36 ± 0
T-SO_2_ (mg/L)	78 ± 1	81 ± 7	71 ± 4	77 ± 6
Color (Abs 420 nm)	0.15 ± 0.01	0.08 ± 0.01	0.08 ± 0.01	0.07 ± 0.01
TPI	8.6 ± 0.0	6.3 ± 0.0	5.6 ± 0.0	7.3 ± 0.0

Total acidity is expressed as g/L of tartaric acid and volatile acidity as g/L of acetic acid. F-SO_2_ denotes free sulfur dioxide (mg/L) and T-SO_2_ denotes total sulfur dioxide (mg/L). TPI denotes the total polyphenol index.

**Table 2:** Experimental design matrix and units.

Matrix	Wood Treatment	Design	Code	Experimental Units
Real wine	Verdejo (V) Albillo (A) Rufete (R) Puesta en Cruz (PC)	No wood (control)	VCT ACT RCT PCCT	4 × 2 = 8
Real wine	Verdejo (V) Albillo (A) Rufete (R) Puesta en Cruz (PC)	Wide-grain (WG) Fine-grain (FG)	VWG-VFG AWG-AFG RWG-RFG PCWG-PCFG	4 × 2 × 3 = 24
Model wine	Synthetic (S)	Wide-grain (WG) Fine-grain (FG)	SWG SFG	2 × 3 = 6

**Table 3:** General chemical characteristics of monovarietal Verdejo, Albillo, Rufete, and Puesta en Cruz wines after 35 days without wood (control) or in contact with the two wood samples.

Wine–Wood	pH	Total Acidity	Volatile Acidity	Alcohol Content	F-SO_2_	T-SO_2_
**Verdejo wines**						
VCT	3.62 ± 0.00 b	4.0 ± 0.1 a	0.40 ± 0.02 a	13.50 ± 0.00 b	30 ± 2 b	75 ± 6 b
VWG	3.60 ± 0.01 a	3.8 ± 0.1 a	0.37 ± 0.05 a	13.40 ± 0.00 a	12 ± 1 a	41 ± 2 a
VFG	3.60 ± 0.00 a	3.9 ± 0.0 a	0.34 ± 0.02 a	13.60 ± 0.00 c	13 ± 1 a	43 ± 5 a
**Albillo wines**						
ACT	3.25 ± 0.00 a	6.5 ± 0.1 b	0.63 ± 0.00 a	13.30 ± 0.00 a	30 ± 2 b	81 ± 7 b
AWG	3.26 ± 0.01 a	6.1 ± 0.0 a	0.69 ± 0.08 a	13.27 ± 0.12 a	15 ± 1 a	65 ± 6 a
AFG	3.26 ± 0.01 a	6.2 ± 0.0 a	0.82 ± 0.02 b	13.33 ± 0.12 a	15 ± 1 a	67 ± 4 a
**Rufete wines**						
RCT	3.24 ± 0.00 b	5.4 ± 0.1 b	0.39 ± 0.04 a	13.60 ± 0.14 b	27 ± 1 b	70 ± 1 b
RWG	3.24 ± 0.00 b	5.6 ± 0.0 c	0.53 ± 0.08 a	13.53 ± 0.12 b	9 ± 2 a	48 ± 7 a
RFG	3.23 ± 0.00 a	5.2 ± 0.0 a	0.41 ± 0.01 a	13.20 ± 0.00 a	7 ± 1 a	53 ± 3 a
**Puesta en Cruz wines**						
PCCT	3.05 ± 0.00 a	7.0 ± 0.1 b	0.37 ± 0.00 a	12.70 ± 0.00 c	34 ± 0 b	76 ± 8 b
PCWG	3.09 ± 0.00 c	6.8 ± 0.0 a	0.40 ± 0.03 a	12.33 ± 0.12 b	20 ± 1 a	45 ± 5 a
PCFG	3.08 ± 0.00 b	6.8 ± 0.0 a	0.40 ± 0.01 a	11.93 ± 0.12 a	18 ± 3 a	55 ± 4 a
**Two-way ANOVA**						
*Wood treatment: F and p values*	9.43 ***	42.35 ***	3.63 **	20.98 ***	383.72 ***	56.42 ***
*Wine variety: F and p values*	38,726.59 ***	4730.74 ***	229.40 ***	307.44 ***	58.04 ***	13.85 ***
*Wood treatment × variety: F and p values*	46.23 ***	20.77 ***	6.91 ***	15.54 ***	1.92	2.00

Data are presented as mean ± standard deviation (SD), with *n* = 2 for control wines and *n* = 3 for wines treated with wood, where *n* represents independent experimental units. Different lowercase letters (a, b, c) indicate statistically significant differences among treatments (control, WG, and FG) within the same wine matrix (Tukey’s HSD test, *p* < 0.05). Total acidity is expressed as g/L of tartaric acid and volatile acidity as g/L of acetic acid. F-SO_2_ denotes free sulfur dioxide (mg/L), and T-SO_2_ denotes total sulfur dioxide (mg/L). Asterisks indicate statistically significant effects in the two-way ANOVA: ** *p* < 0.01, and *** *p* < 0.001.

**Table 4:** Two-way ANOVA for phenolic acids, phenolic aldehydes, and furanic derivatives quantified in wines after 35 days.

Compounds	Wood Treatment: F and *p* Values	Wine Variety: F and *p* Values	Wood Treatment × Variety: F and *p* Values
Chlorogenic acid	2.50	4360.22 ***	3.09 **
Gallic acid	680.21 ***	34.17 ***	2.13
Syringic acid	922.93 ***	14.34 ***	1.76
Vanillic acid	436.08 ***	8.82 ***	8.81 ***
Sinapic acid	84.17 ***	609.50 ***	43.18 ***
p-Coumaric acid	32.53 ***	597.54 ***	190.90 ***
Ferulic acid	15.78 ***	9355.96 ***	204.19 ***
**Total phenolic acids**	873.05 ***	184.79 ***	2.84 *
Vanillin	513.53 ***	2.19	1.90
Coniferaldehyde	296.50 ***	25.72 ***	7.07 ***
Syringaldehyde	1359.48 ***	5.91 **	2.83 ***
Sinapaldehyde	414.87 ***	24.54 ***	7.01 ***
**Total phenolic aldehydes**	865.98 ***	16.41	6.22 ***
5-HMF	701.33 ***	1.44	0.88
5-MF	914.83 ***	11.42 ***	11.36 ***
Furfural	648.75 ***	2.54	4.54 **
**Total furanic derivatives**	1130.24 ***	0.44	0.83

* *p* < 0.1; ** *p* < 0.05; *** *p* < 0.001. *F* and *p* values are based on the independent experimental units (*n* = 2 for control wines and *n* = 3 for wines treated with wood).

**Table 5:** Concentration of phenolic compounds in four monovarietal wines (Verdejo, Albillo, Rufete, and Puesta en Cruz) after 35 days, either without wood (control) or with WG or FG wood contact.

	Catechin	Epicatechin	Protocatechuic Acid	*Trans*-Fertaric Acid	*Trans*-Caffeic Acid	*Cis*-Coutaric Acid	*Cis*-Coumaric Acid	Tyrosol
**Verdejo wines**								
VCT	4.66 ± 0.05 a	2.45 ± 0.17 a	2.33 ± 0.17 a	3.22 ± 0.03 a	0.78 ± 0.02 b	0.85 ± 0.03 a	0.58 ± 0.02 a	2.15 ± 0.02 a
VWG	5.81 ± 0.20 b	2.28 ± 0.03 a	2.17 ± 0.10 a	3.28 ± 0.05 a	0.71 ± 0.02 a	0.80 ± 0.16 a	0.57 ± 0.02 a	2.04 ± 0.02 a
VFG	5.43 ± 0.20 b	2.53 ± 0.10 a	2.36 ±0.10 a	3.23 ± 0.04 a	0.73 ± 0.04 ab	0.89 ± 0.03 a	0.54 ± 0.02 a	2.05 ± 0.02 a
**Albillo wines**								
ACT	2.80 ± 0.08 a	3.17 ± 0.12 a	nd	1.76 ±0.02 a	0.79 ± 0.02 a	1.66 ± 0.02 a	nd	1.53 ± 0.02 a
AWG	3.18 ± 0.16 b	3.21 ± 0.13 a	nd	1.79 ± 0.07 a	0.74 ± 0.03 a	1.84 ± 0.012 b	nd	1.48 ± 0.02 a
AFG	3.13 ± 0.16 b	3.24 ± 0.10 a	nd	1.84 ± 0.02 a	0.76 ± 0.02 a	1.75 ± 0.05 ab	nd	1.47 ± 0.02 a
**Rufete wines**								
RCT	3.16 ± 0.09 a	2.09 ± 0.02 a	1.67 ± 0.04 a	2.30 ± 0.01 a	0.55 ± 0.01 a	1.27 ± 0.01 a	nd	2.77 ± 0.02 b
RWG	3.59 ± 0.15 b	2.24 ± 0.09 b	1.65 ± 0.04 a	3.30 ± 0.03 a	0.54 ± 0.01 ab	1.31 ± 0.02 b	nd	2.64 ± 0.02 a
RFG	3.42± 0.08 ab	2.13 ± 0.05 ab	1.67 ± 0.09 a	3.31 ± 0.03 a	0.53 ± 0.01 a	1.31 ± 0.02 b	nd	2.67 ± 0.02 a
**Puesta en Cruz wines**								
PCCT	3.14 ± 0.11 a	2.26 ± 0.09 a	3.48 ± 0.08 a	2.52 ± 0.02 b	1.51 ± 0.04 b	3.59 ± 0.04 a	0.40 ± 0.01 a	1.90 ± 0.03 b
PCWG	3.05 ± 0.15 a	2.33 ± 0.12 a	3.63 ± 0.03 b	2.44 ± 0.01 a	1.25 ± 0.02 a	3.72 ± 0.06 b	0.40 ± 0.01 a	1.81 ± 0.02 a
PCFG	2.73 ± 0.82 a	2.38 ± 0.11 a	3.61 ± 0.09 ab	2.48 ± 0.03 a	1.28 ± 0.09 a	3.72 ± 0.09 b	0.41 ± 0.02 a	1.81 ± 0.04 a

nd: not detected. Different lowercase letters indicate statistically significant differences among treatments (control, WG, and FG) within the same wine matrix (Tukey’s HSD test, *p* < 0.05). Data are presented as mean ± standard deviation (SD), with *n* = 2 for control wines and *n* = 3 for wines treated with wood, where *n* represents independent experimental units.

**Table 6:** Relative transfer index (RTI) of the different compounds from wood to wine.

	Wide Grain (WG)				Fine Grain (FG)			
	Verdejo Wine	Albillo Wine	Rufete Wine	Puesta en Cruz Wine	Verdejo Wine	Albillo Wine	Rufete Wine	Puesta en Cruz Wine
Vanillic acid	58.2%	30.6%	45.2%	50.7%	59.0%	32.3%	62.0%	52.7%
Syringic acid	92.9%	85.3%	85.5%	76.8%	80.6%	76.6%	85.9%	75.5%
Gallic acid	67.8%	71.5%	71.8%	66.8%	66.0%	70.9%	75.4%	55.3%
**Total acids**	66.3%	68.3%	65.4%	59.1%	60.1%	65.0%	71.1%	56.6%
Vanillin	80.7%	86.3%	85.4%	78.8%	75.9%	74.9%	91.7%	73.6%
Coniferaldehyde	23.4%	24.2%	38.5%	31.9%	24.0%	29.0%	50.0%	37.5%
Syringaldehyde	87.1%	76.2%	93.7%	85.3%	85.4%	76.0%	96.0%	85.7%
Sinapaldehyde	21.1%	21.0%	35.4%	27.0%	25.7%	29.1%	46.0%	34.5%
**Total Aldehydes**	41.0%	39.2%	53.0%	45.2%	43.4%	43.8%	62.1%	50.2%
5-HMF	91.9%	89.0%	88.1%	89.9%	99.4%	101.5%	101.2%	89.6%
5-MF	76.1%	82.4%	88.2%	85.1%	67.2%	76.7%	105.4%	103.1%
Furfural	79.7%	84.3%	88.1%	84.9%	105.0%	112.9%	86.8%	92.0%
**Total furanics**	80.2%	84.3%	88.1%	85.6%	92.2%	100.0%	94.3%	95.4%

RTI values were calculated from the mean concentrations obtained for each real wine and the corresponding model wine under the same experimental conditions; therefore, no dispersion or statistical comparison is reported for this derived index.

**Table 7:** Principal component coefficients.

**PCA 1**	**Component 1**	**Component 2**
Ferulic	−0.016	0.466
Chlorogenic	0.299	0.159
Sinapic	−0.269	0.273
p-Coumaric	0.287	0.184
Vanillic	0.329	0.013
Syringic	0.327	−0.002
Gallic	−0.190	0.379
Vanillin	0.278	0.229
Coniferaldehyde	−0.265	0.289
Syringaldehyde	0.329	0.008
Sinapaldehyde	0.316	−0.075
5-HMF	−0.147	0.436
5-MF	0.237	0.320
Furfural	0.268	0.266
**PCA 2**	**Component 1**	**Component 2**
% *v*/*v*	−0.040	0.328
pH	−0.0013	0.414
F-SO_2_	−0.202	−0.123
T-SO_2_	−0.179	−0.101
TA	−0.022	−0.419
VA	−0.003	−0.275
CI	0.251	0.108
TPI	0.253	0.058
Tartaric esters	0.197	−0.163
Flavonols	0.238	−0.078
Tannins	0.182	−0.221
Ferulic acid	−0.004	0.354
Chlorogenic acid	0.026	0.234
Sinapic acid	0.057	0.351
p-Coumaric acid	0.024	0.119
Vanillic acid	0.257	0.040
Syringic acid	0.268	0.012
Gallic acid	0.243	0.104
Vanillin	0.268	−0.003
Coniferaldehyde	0.251	−0.084
Syringaldehyde	0.267	−0.0256
Sinapaldehyde	0.246	−0.077
5-HMF	0.255	0.029
5-MF	0.265	−0.059
Furfural	0.258	−0.002
**PCA 3**	**Component 1**	**Component 2**
% *v*/*v*	−0.220	0.188
pH	−0.276	0.209
F-SO_2_	0.079	−0.183
T-SO_2_	0.126	−0.136
TA	0.233	−0.274
VA	0.063	−0.310
CI	0.001	0.327
TPI	−0.083	0.124
Tartaric esters	0.221	−0.022
Flavonols	0.233	0.145
Tannins	0.235	−0.067
Ferulic acid	−0.256	0.165
Chlorogenic acid	−0.033	0.278
Sinapic acid	−0.278	0.128
p-Coumaric acid	−0.175	−0.117
Vanillic acid	0.086	0.325
Syringic acid	0.139	0.322
Gallic acid	−0.297	0.0538
Vanillin	0.142	0.262
Coniferaldehyde	0.269	0.141
Syringaldehyde	0.205	0.235
Sinapaldehyde	0.275	0.212
5-HMF	−0.215	−0.023
5-MF	0.290	0.118
Furfural	−0.021	0.011

## Data Availability

The original contributions presented in the study are included in the article and Supplementary Materials; further inquiries can be directed to the corresponding author.

## Supplementary Materials

The following supporting information can be downloaded at: https://www.mdpi.com/article/10.3390/foods15183176/s1, Table S1: HPLC-DAD calibration and chromatographic parameters for the phenolic and furanic compounds analyzed in wood extracts and wines.
