# Supplementary material for: Phenolic Transfer and Dissolved-Oxygen Dynamics in Different White Wines Treated with Two Quercus alba Wood Samples with Contrasting Grain Widths
Source: Foods. 2026 Sep 8;15(18):3176. doi: 10.3390/foods15183176 (PMC13605322; doi:10.3390/foods15183176)
Supplement: Supplementary file 1 [file foods-15-03176-s001.zip › foods-4493363-supplementary.pdf]

Table S1. HPLC-DAD calibration and chromatographic parameters for the phenolic and furanic compounds analyzed in wood extracts and wines.

Compounds	λ (nm)	Calibration range (mg/L)	RT	Regression equation	R^2	LOD	LOQ
Gallic acid	280	0.34-83.82	12.6	y = 159.46x - 85.23	0.9995	0.09	0.34
Syringic acid	280	0.24-60.72	54	y = 153.11x - 40.492	0.9997	0.06	0.24
Vanillic acid	254	0.23-58.74	49	y = 164.2x - 48.947	0.9997	0.06	0.23
p-Cumaric acid	320	0.16-26.69	69	y = 291.97x - 88.174	0.9979	0.05	0.16
Sinapic acid	320	0.09-37.76	81	y = 230.64x - 7.8578	0.9999	0.02	0.09
Chlorogenic acid	320	0.08-2.03	47	y = 137.92x - 7.31	0.9985	0.02	0.08
Ferulic acid	320	0.02-6.64	76	y = 270.24x - 9.3708	0.9995	0.01	0.02
Vanillin	280	0.18-29.78	62.6	y = 219.03x - 6.8311	0.9997	0.05	0.18
Syringaldehyde	320	0.14-60.12	71	y = 169.8x + 14.252	0.9998	0.04	0.14
Furfural	280	0.80-133.84	26.7	y = 444.22x + 533.95	0.999	0.09	0.80
5-MF	280	0.63-105.73	51.3	y = 46.299x - 20.67	0.9998	0.17	0.63
5-HMF	280	0.24-80	21.3	y = 378.71x - 17.659	1	0.04	0.24
Catechin	280	0.23-59.16	44.3	y = 30.716x – 32.995	0.9967	0.07	0.23
Epicatechin	280	0.19-32.77	59.6	y = 50.413x – 20.381	0.9989	0.06	0.19
Protocatechuic acid	254	0.24-60.54	23.4	y = 152.23x – 58.58	0.9995	0.07	0.24
trans-Caffeic acid	340	0.12-20.62	49.3	y = 204.64x – 60.37	0.9975	0.03	0.12

LOD: limit of detection LOQ: limit of quantification
